# Use of a microfluidic platform to uncover basic features of energy and environmental stress responses in individual cells of *Bacillus subtilis*

**DOI:** 10.1371/journal.pgen.1006901

**Published:** 2017-07-20

**Authors:** Matthew T. Cabeen, Jonathan R. Russell, Johan Paulsson, Richard Losick

**Affiliations:** 1 Department of Molecular and Cellular Biology, Harvard University, Cambridge, Massachusetts, United States of America; 2 Department of Systems Biology, Harvard Medical School, Boston, Massachusetts, United States of America; University of Chicago, UNITED STATES

## Abstract

Bacteria use a variety of stress-sensing systems to sense and respond to diverse stressors and to ensure their survival under adverse conditions. The gram-positive bacterium *Bacillus subtilis* responds to energy stress (ATP depletion) and to environmental stressors using two distinct stress-sensing pathways that converge on the alternative sigma factor σ^B^ to provoke a general stress response. Past efforts to study the σ^B^ stress response in bulk culture and on agarose pads were unable to visualize the responses of individual cells under tightly controlled conditions for extended periods of time. Here we use a microfluidics-based strategy to discern the basic features of σ^B^ activation in single cells in response to energy and environmental stress, both immediately upon stressor exposure and for tens of generations thereafter. Upon energy stress at various levels of stressor, cells exhibited fast, transient, and amplitude-modulated responses but not frequency modulation as previously reported. Upon environmental stress, which is mediated by the stressosome complex, wild-type cells primarily exhibited a transient and amplitude-modulated response. However, mutant cells producing only one of the four paralogous RsbR stressosome proteins showed striking and previously unseen differences. Whereas RsbRA-only cells mimicked the wild type, RsbRC-only cells displayed a slower but sustained overall response composed of repeated activation events in single cells.

## Author summary

All living things must sense and respond to stress in order to survive. Because bacteria are often subjected to rapidly changing conditions in nature, they have evolved stress-response mechanisms that are poised to respond to harsh environmental conditions. Many of the proteins that mediate bacterial stress responses are known, but technical limitations have made it difficult to discern how individual cells respond to stress at short and long time scales. By using a microfluidic device in which we can continuously observe individual bacteria as we expose them to different stresses, we have overcome previous limitations and uncovered basic features of bacterial stress responses. Knowledge of these features will help us to understand how different stress-response profiles may benefit cells under stressful circumstances and how cell-to-cell variability may enhance the survival of a population of cells experiencing harsh conditions. Our results from a relatively simple bacterial model system may also yield insights into how higher cells effectively respond to stress, as many stress-response principles are broadly conserved throughout biology.

## Introduction

**Fig 1:**
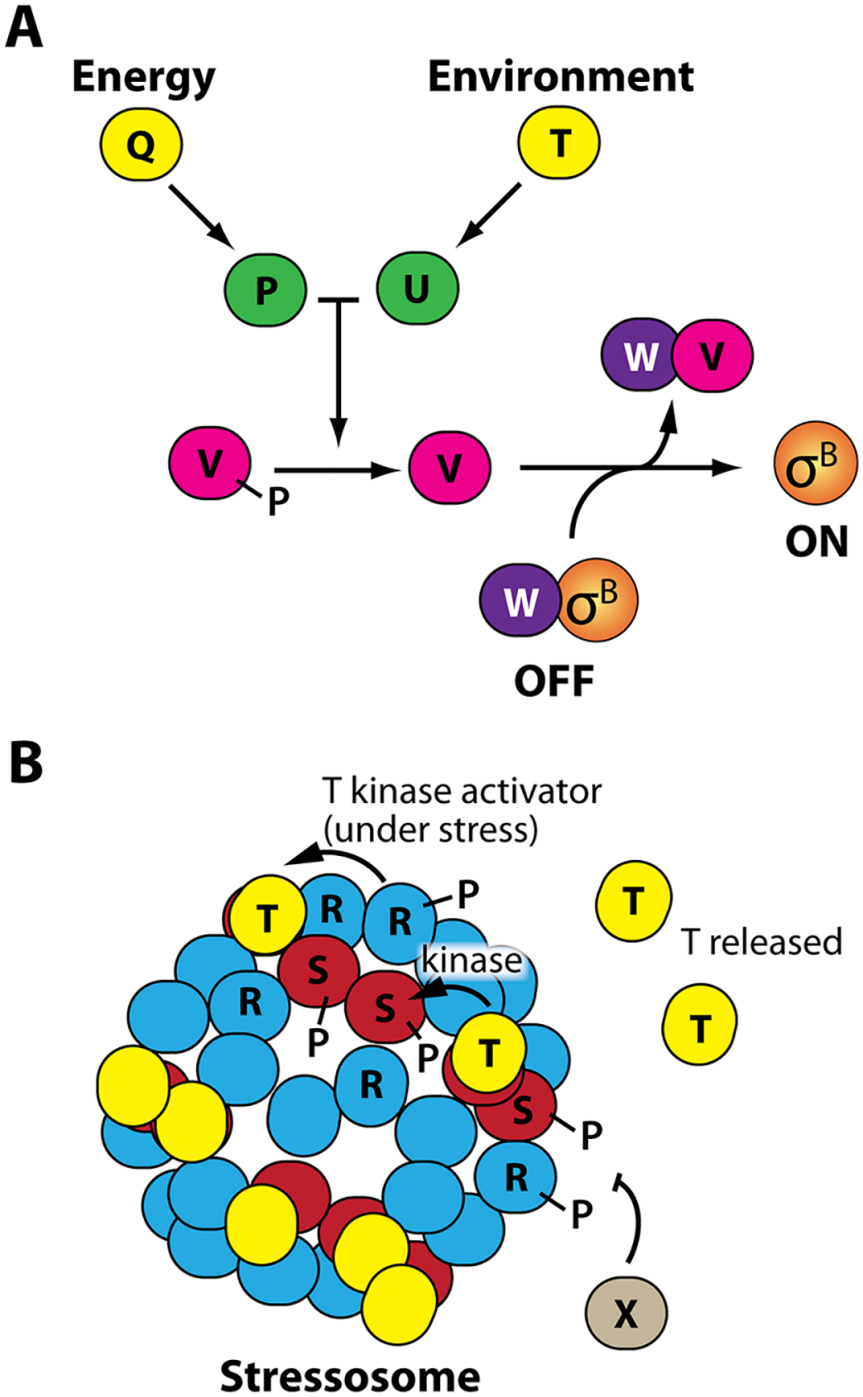
Stress sensing in *B*. *subtilis*. **A.** Energy and environmental stress activate distinct branches of the stress-sensing pathway. Energy stress activates RsbP (P) via an unknown process requiring RsbQ (Q), whereas environmental stress activates RsbU (U) via the RsbU activator RsbT (T). RsbP and RsbU are both phosphatases that dephosphorylate the anti-anti-sigma-factor RsbV (V), causing the anti-sigma factor (and RsbV kinase) RsbW (W) to switch partners and thereby free σ^B^ to direct the transcription of the general stress response regulon. **B.** Environmental stress is sensed by cytoplasmic stressosomes, large, well-characterized protein complexes consisting of RsbR (R) and RsbS (S) proteins that capture RsbT (T) proteins to keep stress signaling in an off state. Upon stressor exposure, RsbR activates the kinase activity of RsbT, leading to the phosphorylation of RsbR and RsbS proteins while RsbT is freed from the stressosome to activate RsbU. The phosphatase RsbX (X) dephosphorylates RsbR and RsbS to return the system to its resting state.

Microorganisms respond to stressful conditions by activating genes that facilitate cell survival. These stress responses often involve the activation of an alternative sigma factor, such as in *Escherichia coli* the heat shock factor σ^32^, the cell envelope stress factor σ^E^, and the general stress-response factor σ^S^ [1, 2]. In the gram-positive bacterium *Bacillus subtilis*, the general stress response is mediated by the alternative sigma factor σ^B^ [3, 4]. We chose σ^B^ as the subject of this investigation, as its relatively well-understood signaling pathway facilitates specific manipulations. Under non-stress conditions, σ^B^ is held inactive by the anti-sigma factor RsbW, which binds to σ^B^ and prevents it from binding to RNA polymerase (Fig 1) [5]. Upon stress, the anti-anti-sigma factor RsbV binds to RsbW, thereby freeing σ^B^ to bind to RNA polymerase and activate stress-response genes [6–8]. RsbV is itself regulated at the level of phosphorylation and is only able to bind to the anti-sigma factor RsbW in its dephosphorylated state [9, 10]. Although σ^B^ activity responds to relatively low levels of stress that have minimal effects on cell growth, σ^B^ has an important role in cell survival at higher stress levels [11, 12].

Activation of σ^B^ is controlled by two distinct upstream pathways that detect different types of stress but converge on the common activation system described above (Fig 1) [13]. When cells undergo energetic or nutritional stress (i.e., ATP depletion), the proteins RsbQ and RsbP are both required to sense this stress by an unknown mechanism, but it is RsbP that then dephosphorylates RsbV [14, 15]. Environmental stress signaling, which is activated by such factors as ethanol, salt, acid, and heat, instead occurs via a more complex pathway that activates RsbU, which like RsbP desphosphorylates RsbV [10, 16–19]. The pathway begins with the stressosome, a 1.8-MDa complex primarily composed of RsbR and RsbS proteins [20–22]; there are an estimated 10–20 stressosomes per cell [23]. When cells are unstressed, RsbT proteins are bound to stressosomes via RsbS, keeping RsbT inactive. Environmental stress is thought to be sensed by the RsbR proteins, a contention supported by their N-terminal non-heme globin domains that extend out from the stressosome core [23–25]. *B*. *subtilis* produces five RsbR paralogs (further discussed in the Results below) that are thought to be mixed among stressosomes [21]. Upon stressor exposure, RsbR activates the kinase activity of RsbT, which then phosphorylates RsbS and RsbR during its release from the stressosome [26]. The freed RsbT then activates RsbU phosphatase activity as described above [10, 16–19]. The phosphorylation of RsbR and RsbS is reversed by the phosphatase RsbX [10, 27] to recapture RsbT and reset the environmental stress-response system.

Because of their small size, bacterial cells experience rapid changes in their natural local environments, and their survival depends on their ability to mount stress responses rapidly and with appropriate magnitude and duration. Stress responses also represent a circumstance in which heterogeneity across a population can be particularly important, as cell-to-cell variation can permit "bet hedging", in which a community of cells can benefit from some cells being better adapted to unpredictable future changes in local conditions. Therefore, an understanding of the basic features of bacterial stress responses requires immediate observation of cells upon stress exposure, so as to reveal the speed and magnitude of the initial response; observations of individual cells, so as to evaluate cell-to-cell variations across a population; and long-term observation, so as to uncover any response trends beyond the initial response to stressor onset. However, these basic features are not yet well understood, primarily because of technical limitations to previously employed methods. Classic bulk-culture experiments in flasks have a limited observational window (typically under one hour) and do not yield single-cell information. Agarose pad-based experiments give single-cell data, but it is difficult to observe immediate responses to stress onset, and the observational window is limited. With both methods, cellular metabolism continuously changes the local environment in uncontrolled ways.

Here, we use a microfluidics-based approach [28], which we previously adapted for *B*. *subtilis* [29], to observe bacterial stress responses under constant exponential-phase growth conditions. This experimental strategy permits us to add a defined stressor to the medium flow and then to observe lineages of single cells both as they first encounter the stressor and over the course of many cell generations thereafter as they adapt to the presence of the stressor; hence, our observational window is much longer than for bulk culture- or agarose pad-based approaches. The microfluidic device also maximizes spatial and temporal uniformity so as to highlight true cell-to-cell heterogeneity. Using this approach, we discern the basic features of the *B*. *subtilis* σ^B^ stress response to different levels of energy and environmental stress. We also find previously unappreciated distinctions among the environmental stress-response profiles mediated by the four paralogous RsbR proteins encoded by the *B*. *subtilis* genome.

## Results

### The energy-stress response is transient and amplitude-modulated

**Fig 2:**
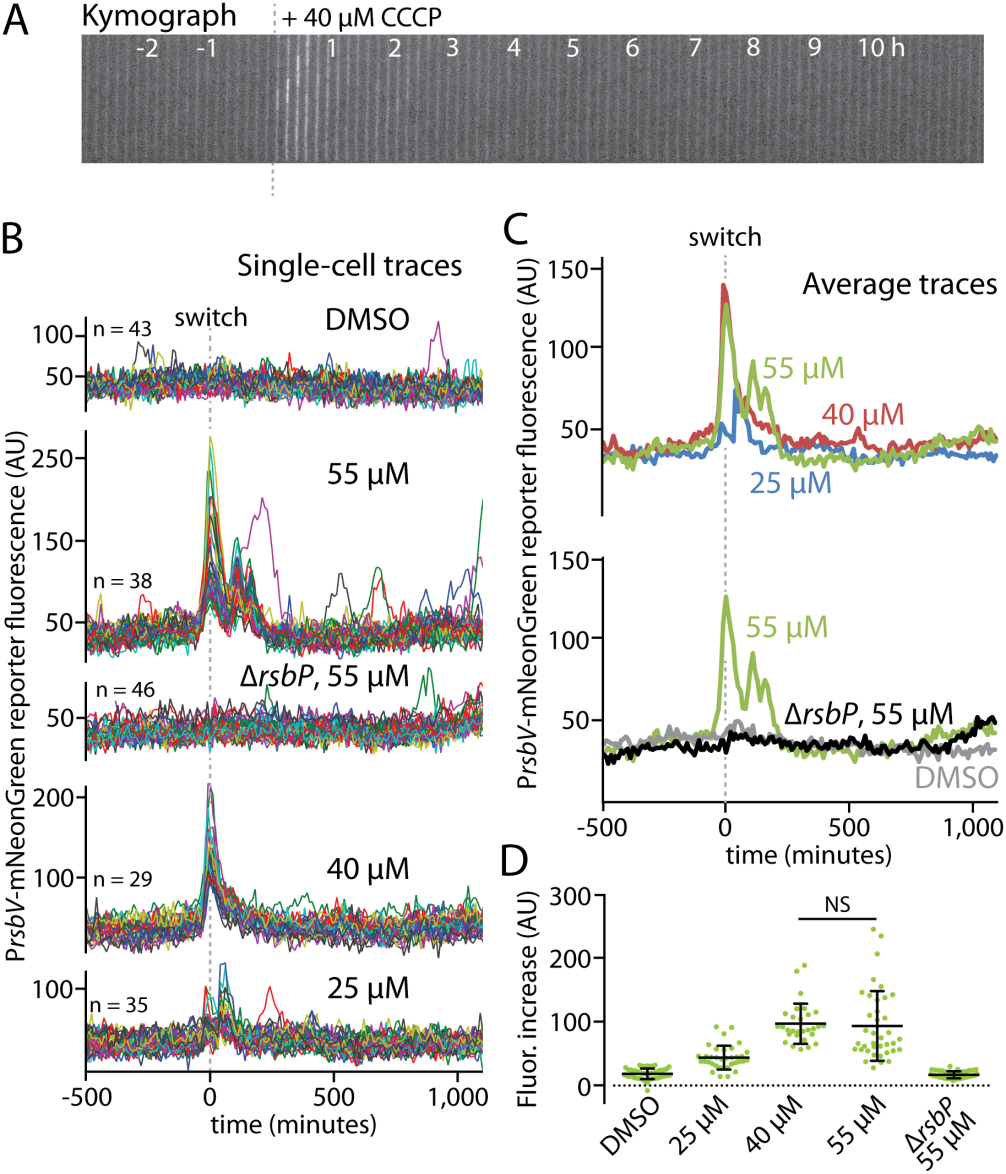
Energy stress elicits an amplitude-modulated response. **A.** A kymograph is shown of an MTC1801 cell lineage growing in a microfluidic channel before and after the addition of 40 μM CCCP (dashed line) to induce energy stress via ATP depletion. **B.** Single-cell intensity traces of a stress-responsive P_*rsbV*_-mNeonGreen reporter before and after (dashed line) the addition of CCCP at the indicated concentrations. DMSO was used as a vehicle control for the medium switch, and a Δ*rsbP* mutant (MTC1906) controlled for non-energy-stress responses. The traces were manually curated to eliminate cell lineages with cell-death events or other anomalies; comparisons of mean traces for curated and uncurated populations are shown in S2 Fig. **C.** Overlaid average intensity traces of the cell populations in **B**, showing different CCCP concentrations (top) and the controls (bottom). **D.** Distributions of signal increases in single cells from before stress exposure to their maximal peak values. NS, not significant; all other pairwise comparisons (except for DMSO vs. Δ*rsbP*) were significant (*p* < 4 x 10^−6^).

We began by using the microfluidic platform to investigate the response of individual cells to energy stress. A previous investigation based on the use of agarose pads gave rise to a frequency-modulation model that posited that energy-stress responses were pulsatile, with pulses getting more frequent (but not greater in amplitude) as stressor levels increased [30]. Such a frequency-modulated response might in principle create a broad distribution of cellular states in which only some cells would be responding at any given time; such heterogeneity across a cell population might then represent a bet-hedging strategy [30]. Because the previously reported pulse frequency was on the order of a few hundred minutes, only 2–3 pulses were observable over the course of an experiment. Also, the use of agarose pads only allowed observations to be made in a steady-state condition, well after cells had already been exposed to the stressor. We reasoned that our microfluidic platform would be an ideal system to observe frequency modulations, not only because we could observe cell lineages for longer (and hence see a greater number of response pulses), but also because we could add the stressor to unstressed cells and watch the initial response to stress in addition to observing the steady state behavior of cells under stress. We reasoned that the initial response of cells to the sudden introduction of an energy stressor might be relatively synchronous, with the amplitude of the response tailored to the level of stress. Indeed, earlier bulk-culture studies showed initial responses in tens of minutes but did not examine the response trends for longer than 60 minutes or across different stressor concentrations [31, 32]. We chose as an energy stressor the oxidative phosphorylation inhibitor CCCP (carbonyl cyanide *m*-chlorophenyl hydrazone), a direct ATP-depleting agent that elicited a relatively fast and robust energy-stress response in previous work [31, 32]. Our results showed a clear, rapid, and transient energy-stress response upon exposure to 40 μM CCCP (Fig 2A and S1 Movie).

Switching growing cells into medium containing only the dimethyl sulfoxide (DMSO) vehicle that we used to deliver the CCCP induced no visible response, whereas exposing cells to increasing concentrations of CCCP elicited progressively greater response amplitudes (Fig 2B–2D and S1–S4 Movies). To ensure that the observed responses did not result from crosstalk between the energy and environmental stress signaling branches, we used a Δ*rsbP* mutant to inactivate energy signaling. Importantly, the Δ*rsbP* mutant showed no response, even at the highest tested CCCP concentration (Fig 2B–2D and S5 Movie). Moreover, the amplitude of the response appeared to plateau above 40 μM CCCP, as 55 μM CCCP did not induce a significantly stronger response (Fig 2D) but did show additional, closely spaced response peaks (Fig 2B and 2C and S3 Movie). This broadening of the overall response peak once the maximal response amplitude has been reached may represent a strategy to enhance energy-stress protection at near-toxic stressor levels (in preliminary experiments, exposure to CCCP concentrations above 60 μM resulted in widespread cell death). Strikingly, and in contrast to a previous report [30], we observed virtually no additional long-term response pulses after the initial response to CCCP onset, even when observing cells for over 1,000 minutes in the presence of CCCP (Fig 2B). We saw occasional peaks in cell lineages exposed to 55 μM CCCP, but such peaks were extremely infrequent, much weaker than the initial response peak, and absent from cells treated with lower CCCP concentrations (Fig 2B). We conclude that, under our experimental conditions, the energy-stress response is amplitude-modulated rather than being frequency-modulated, suggesting that cells prioritize a more-stereotyped initial response to energy stressor onset over more broadly distributed, sustained responses in a stressor-exposed state.

Finally, we investigated the response to mycophenolic acid, which was used as an energy stressor in the study of Locke et al. [30]. S1 Fig shows that cells responded strikingly slower to mycophenolic acid than was seen in the experiments with CCCP (190 min to reach a half-maximal response for 60 μg/ml of mycophenolic acid versus 20 min for 40 μM CCCP). Also, the response to mycophenolic acid was sustained for the duration of the experiment, unlike the response to CCCP, which was transient. These differences may reflect the fact that mycophenolic acid acts indirectly to trigger energy stress by inhibiting GTP synthesis rather than directly to block ATP production as in the case of CCCP [32]. In any event, although the sustained response exhibited some noise in single cells (S1B Fig), we saw little evidence of pulsatile behavior. While we cannot exclude the possibility that slight or fast pulsing was obscured by fluorescent protein maturation, we conclude that any pulsatile component is not a major contributor to the overall energy-stress responses we observed.

### Ethanol stress provokes both transient and sustained responses depending on stressor concentration

**Fig 3:**
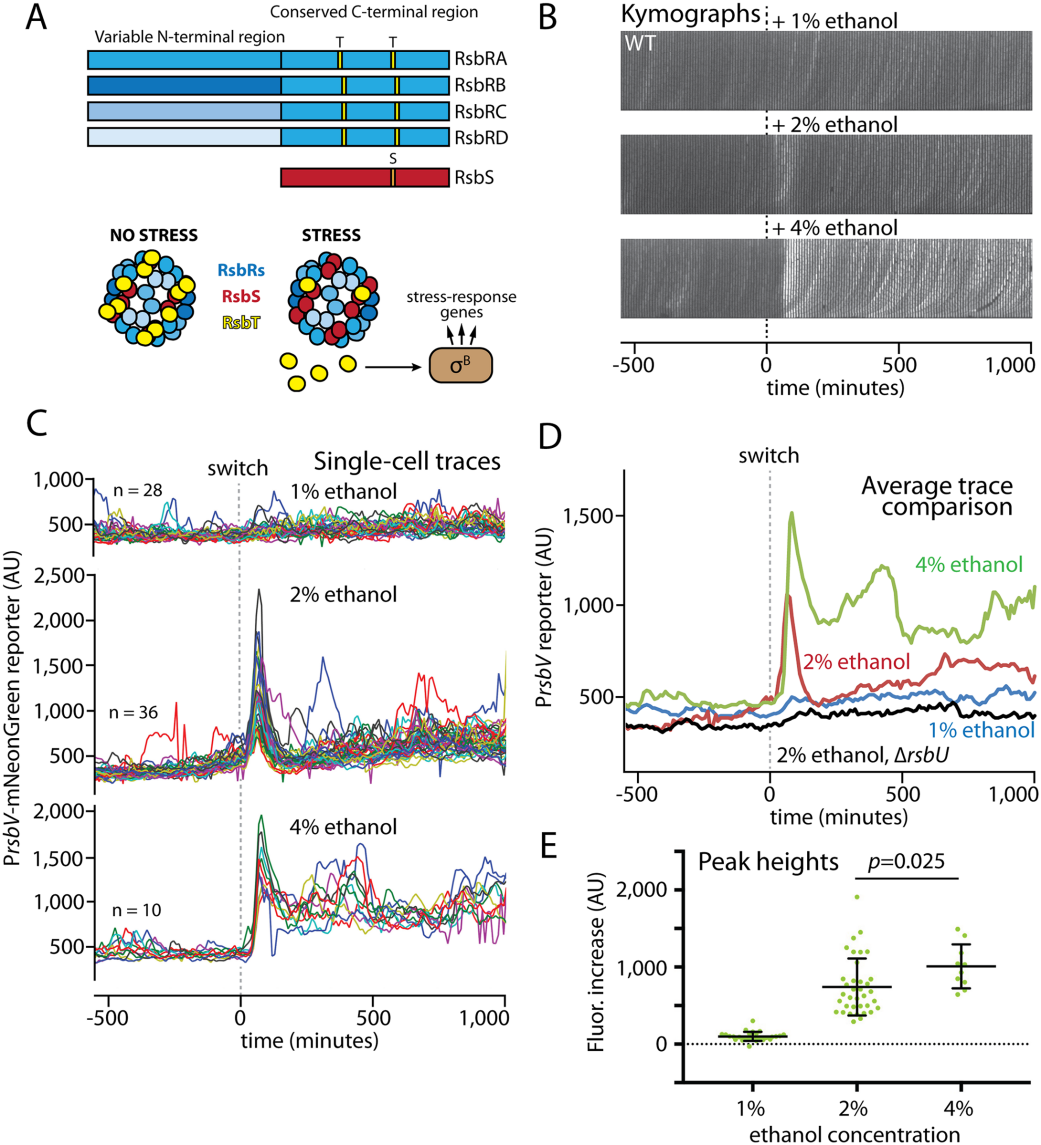
Environmental stress levels elicit different response profiles in wild-type cells. **A.** Schematic of the four RsbR paralogs and RsbS, showing variable and conserved domains with RsbT-phosphorylatable S/T residues (orange). Wild-type cells presumably have mixed populations of RsbR proteins (different shades of blue) in their stressosomes. Stress leads to RsbT release and subsequent σ^B^ activation (also see Fig 1). **B.** Kymographs of single representative wild-type (MTC1801) cell lineages growing in microfluidic channels before and after addition of the indicated ethanol concentrations (dashed line) to induce environmental stress. **C.** Single-cell intensity traces of a stress-responsive P_*rsbV*_-mNeonGreen reporter before and after (dashed line) the addition of ethanol at the indicated concentrations. **D.** Overlaid average intensity traces of the cell populations in **C** showing different ethanol concentrations, together with the average trace of a control Δ*rsbU* (environmental stress-negative) strain upon 2% ethanol exposure. The traces were manually curated to eliminate cell lineages with cell-death events or other anomalies; comparisons of mean traces for curated and uncurated populations are shown in S3 Fig. **E.** Distributions of signal increases in single cells from before stress exposure to their maximal peak values. All unmarked pairwise comparisons were significant (*p* < 3 x 10^−6^).

We next turned to the environmental branch of the stress-response pathway. In contrast to the relatively simple energy-stress branch consisting only of the RsbQ and RsbP proteins (Fig 1), environmental stress is mediated by large stressosome complexes consisting of RsbR proteins along with RsbS and RsbT (Fig 3A) [20–23]. As described above, the stressosome functions by sequestering RsbT until stress is sensed, at which time RsbT is freed to activate the downstream steps of stress signaling (Fig 3A) [10]. Notably, *B*. *subtilis* encodes four paralogous RsbR proteins known as RsbRA, RsbRB, RsbRC, and RsbRD [24]. A fifth paralog, YtvA, is a blue-light sensor [33, 34] that differs from the other four in that it evidently cannot form stressosomes on its own *in vivo* [22, 24, 35]; because it had only minor effects on the magnitude and not the shape of RsbRA-mediated stress responses in preliminary experiments, we used a *ytvA*-deleted strain background for all the experiments in this study to avoid spurious activation by fluorescence imaging. The RsbR paralogs share with RsbS conserved C-terminal STAS domains that compose the stressosome core, whereas the more-divergent N-terminal regions form non-heme globin dimers that extend out from the stressosome core [23, 24]. Wild-type stressosomes are regarded as containing stochastic mixtures of the RsbR paralogs [21], as indicated by different shades of blue in Fig 3A.

We first sought to observe the responses of cells to different concentrations of ethanol, a classic environmental stressor well-known to provoke a strong general stress response. Challenging cells with 1% ethanol did not induce a readily discernible response, whereas 2% ethanol provoked a strong and transient response spike that was synchronous across the cell population (Fig 3B and 3C and S7 and S8 Movies). This response profile resembled the canonical response previously seen in bulk-culture experiments [16, 22, 24] and in microcolonies on agarose pads or under microfluidic conditions [36], suggesting that growth in our microfluidic device does not appreciably change the general principles of the cellular response to environmental stress. The response was also specific to the environmental-stress pathway, as cells deleted for *rsbU* showed a minimal response to 2% ethanol (S10 Fig and S9 Movie). When we further challenged wild-type (*rsbU*^+^) cells with 4% ethanol, we observed a similar synchronous response spike that was only slightly stronger than that elicited by 2% ethanol (Fig 3B–3E and S10 Movie), implying amplitude modulation resembling that of the energy-stress response (Fig 2) and in agreement with previous results from bulk culture [16] and agarose pads [36]. Moreover, there was a sharp increase in the mean output amplitude (approximately 8-fold) in response to a twofold change in the input stressor (from 1% to 2% ethanol; Fig 3E), in accord with an ultrasensitive, switch-like system. The modest further increase at 4% ethanol suggested that the system was nearing saturation. Exposure to 4% ethanol also increased the rate of cell death, resulting in a smaller number of high-quality cell lineages (Fig 3C). Notably, 4% ethanol, in contrast to 2% ethanol, elicited a sustained response that endured for the duration of the experiment, as individual cells seldom returned to their pre-stress reporter levels (Fig 3B–3D and S10 Movie). As the cells were continuously exposed to ethanol after the medium switch, this result suggests that wild-type cells experience a relative insensitivity to ethanol stress after their initial exposure and that a higher ethanol concentration (e.g., 4%) can partially overcome the insensitivity to provoke a more-sustained stress response. This sustained response at 4% ethanol represents a previously unappreciated response feature that was only uncovered by the long-term observation of cells well after the initial response peak. As with the peak-broadening that we observed at high levels of energy stress (Fig 2B and 2C), this sustained response may enhance cellular stress tolerance once the maximum response amplitude is reached.

### Each RsbR paralog mediates a distinct response to ethanol stress

**Fig 4:**
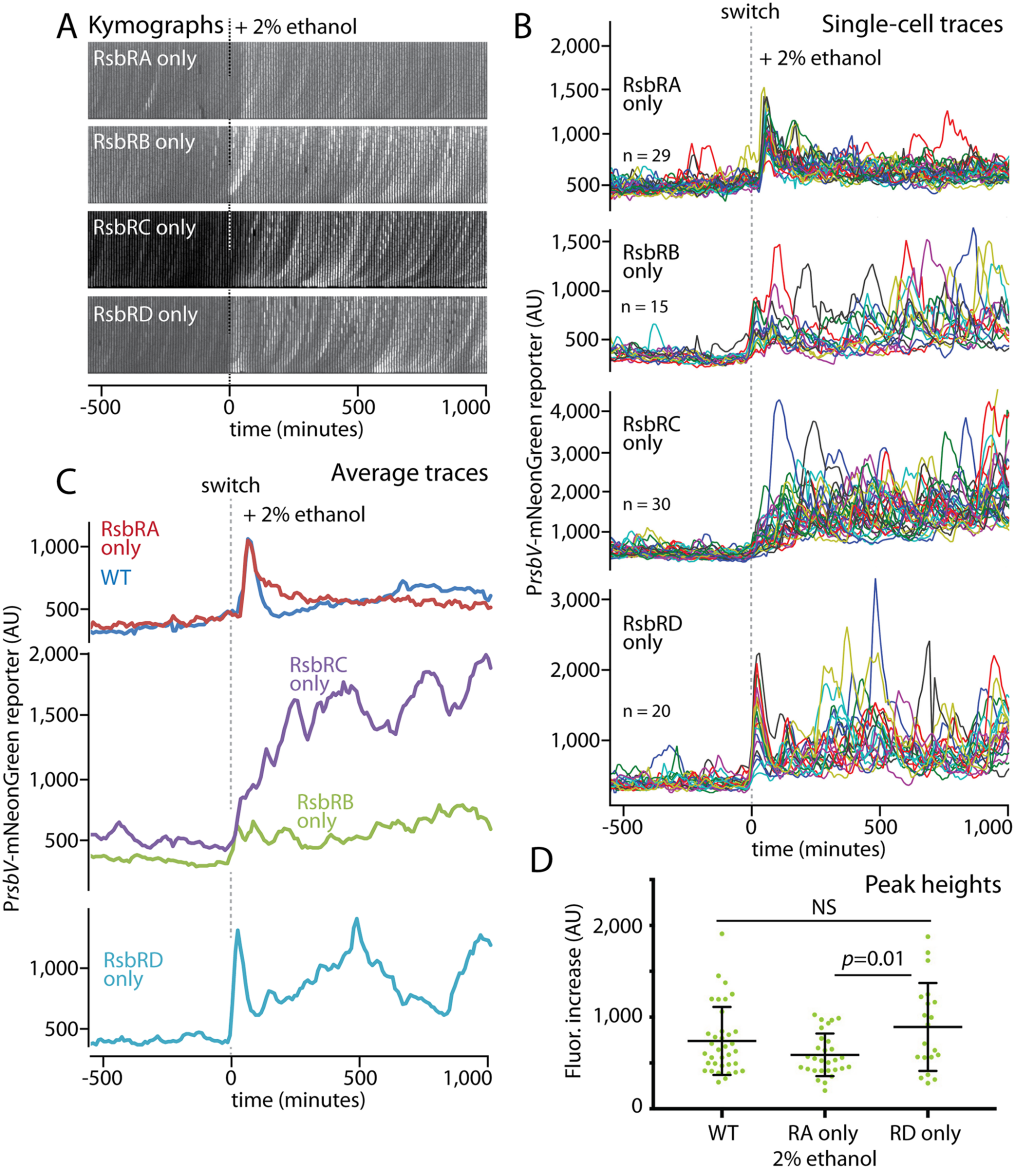
The four RsbR paralogs exhibit distinct response profiles in response to an identical stress. **A.** Kymographs of individual cell lineages of strains containing RsbRA, RsbRB, RsbRC, or RsbRD as the only RsbR in the cell (MTC1761, 1763, 1765, and 1767, respectively). Ethanol was added at t = 0 (dashed lines). **B.** Single-cell intensity traces of a stress-responsive P_*rsbV*_-mNeonGreen reporter the single-RsbR strains responding to the addition of 2% ethanol (dashed line). **C.** Average intensity traces of the cell populations shown in **B**, grouped to highlight similarities and differences. The traces were manually curated to eliminate cell lineages with cell-death events or other anomalies; comparisons of mean traces for curated and uncurated populations are shown in S4 Fig. **D.** Distributions of signal increases in single cells from before stress exposure to their maximal peak values; only strains showing a sharp and transient initial response were evaluated. NS, not significant.

The coexistence of four RsbR paralogs in the stressosome with their variable N-terminal regions extending from the stressosome core makes it attractive to hypothesize that each paralog fulfils a distinct cellular function. For instance, the RsbRs might sense different specific stressors or respond with different sensitivity. However, it has been a persistent challenge to identify functional differences among the RsbRs [22, 35]. We thus asked whether individual RsbRs might display different responses, reasoning that the long observation window, constant environment, and single-cell resolution of our microfluidic platform might uncover previously unappreciated differences. To examine each RsbR paralog individually, we constructed sets of triple-deletion strains that would produce only one of the four RsbR proteins and then challenged these strains with 2% ethanol. We observed striking differences in their stress-response profiles. Cells containing only RsbRA exhibited a transient and synchronous response spike that almost perfectly resembled the wild-type response (Fig 4A–4C and S11 Movie). In sharp contrast, RsbRC showed a slower, progressive response that reached a high average level and was sustained for the duration of the experiment (Fig 4A–4C and S12 Movie). The response mediated by RsbRB resembled the RsbRC response but was substantially weaker (Fig 4A–4C and S13 Movie). RsbRD, meanwhile, showed a hybrid response composed of an initial response spike that resembled the wild-type response in magnitude (Fig 4D and S14 Movie) and was then followed by a sustained response. Single-cell traces revealed that the sustained mean responses observed for the RsbRB-, RsbRC-, and RsbRD-only populations were composed of repeated stress-activation peaks in single cells (Fig 4A and 4B; S7 and S8 Figs highlight the greater standard deviations and coefficients of variance for these populations). Together, our results reveal that the RsbR paralogs have different response characteristics when challenged with an identical 2% ethanol stress. The different RsbRs differed in the speed of their initial response, in their overall and single-cell response magnitudes, and in the duration of the overall response. The repeated σ^B^ activity peaks in single cells during sustained responses give rise to a broad activity distribution over a cell population, resembling the proposed bet-hedging strategy that would be facilitated by frequency-modulated responses [30].

### RsbRA-only and RsbRC-only cells display distinct amplitude-modulated responses

**Fig 5:**
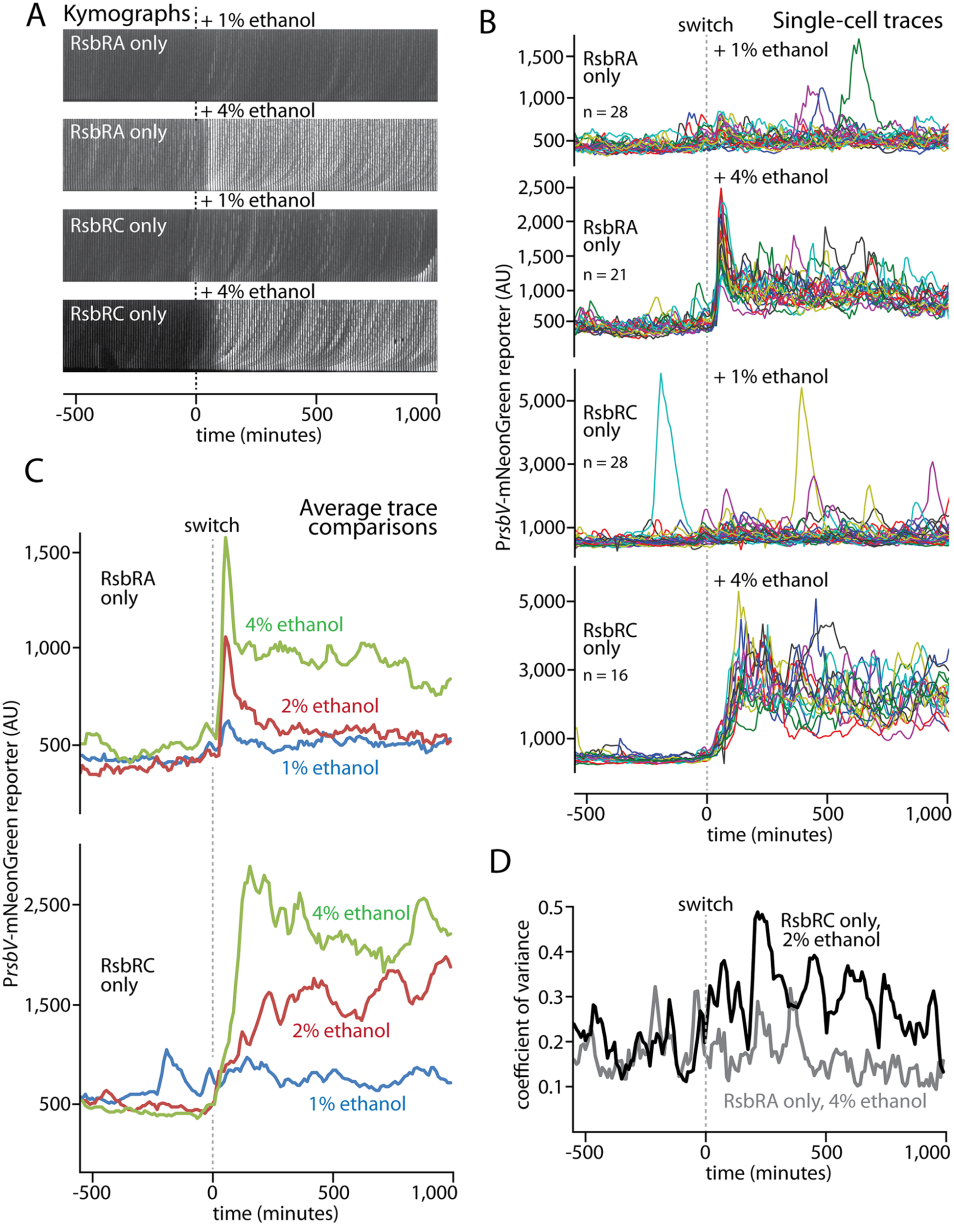
Responses of RsbRA- and RsbRC-only cells to different ethanol concentrations. **A.** Kymographs of individual cell lineages of strains containing RsbRA or RsbRC as the only RsbR in the cell (MTC1761 or 1765, respectively). Ethanol was added at the indicated concentrations at t = 0 (dashed lines). **B.** Single-cell intensity traces of a stress-responsive P_*rsbV*_-mNeonGreen reporter before and after (dashed line) the addition of ethanol at the indicated concentrations with the indicated strains. **C.** Overlaid average intensity traces of the cell populations in **B** showing different ethanol concentrations. The traces were manually curated to eliminate cell lineages with cell-death events or other anomalies; comparisons of mean traces for curated and uncurated populations are shown in S5 Fig. **D.** Coefficient-of-variance plot comparing the RsbRA-only strain at 4% ethanol with the RsbRC-only strain at 2% ethanol. The cell populations in **B** were used for the computation.

Having observed distinct response profiles for each of the four RsbR paralogs, we focused our attention on RsbRA and RsbRC, as these paralogs showed the greatest contrast in their response profiles (and are known to be produced at similar levels [37]). Whereas RsbRA-only cells responded to ethanol stress with a fast, sharp, and transient response, RsbRC-only cells displayed a slower initial response that progressively reached a higher mean level and was then sustained for the duration of ethanol exposure (Fig 4A–4C). We thus asked how these response profiles would change in response to lower or higher concentrations of ethanol. When challenged with 1% ethanol, both strains showed a modest response (Fig 5A–5C and S15 and S16 Movies), in accord with our results for wild-type cells (Fig 3B and 3C). RsbRA-only cells showed a small but detectable spike in the population average trace (Fig 5C and S5B Fig), whereas the RsbRC-only strain responded with a broadening of the variation across the cell population that was caused by more-frequent response events in individual cells (Fig 5B and S7 Fig). Hence, even at stress levels close to the response threshold, RsbRA and RsbRC maintain their strikingly distinct response profiles but at substantially lower amplitudes. The modest responses of both strains to 1% ethanol also support the idea that both RsbRA and RsbRC appear to exhibit some degree of ultrasensitivity between 1% and 2% ethanol.

When stressed with 4% ethanol, both strains also largely preserved their characteristic response profiles but substantially increased their response magnitudes. RsbRC-only cells again manifested a progressively increasing response that was faster and reached a higher sustained level than with 2% ethanol (Fig 5C and S17 Movie). In contrast, RsbRA-only cells showed a transient response spike that was notably stronger than that elicited by 2% ethanol and that was followed by a clear sustained response (Fig 5C, S5B Fig and S18 Movie) characterized in single-cell traces by greater variability and more-frequent response spikes (Fig 5B). The sustained phase of the RsbRA response in 4% ethanol thus resembled the sustained RsbRC response yet was clearly distinguished by its narrower variability across the cell population, as indicated by coefficient-of-variance plots (Fig 5D and S8 Fig). The long-term sustained responses of the RsbRA- and RsbRC-only strains at 4% ethanol were only distinguishable thanks to the long observation times and single-cell resolution of a microfluidics-based approach. Meanwhile, the immediate response of RsbRA-only cells showed strong amplitude modulation with different ethanol concentrations (S5B Fig), partially mimicking the wild type but being distinguished by its greater increase in magnitude between 2% and 4% ethanol. Thus, both the RsbRA- and RsbRC-only responses to different ethanol concentrations appear to be less switch-like than that of the wild-type.

### RsbRA-only and RsbRC-only cells show contrasting single-cell response profiles and wide response variability

**Fig 6:**
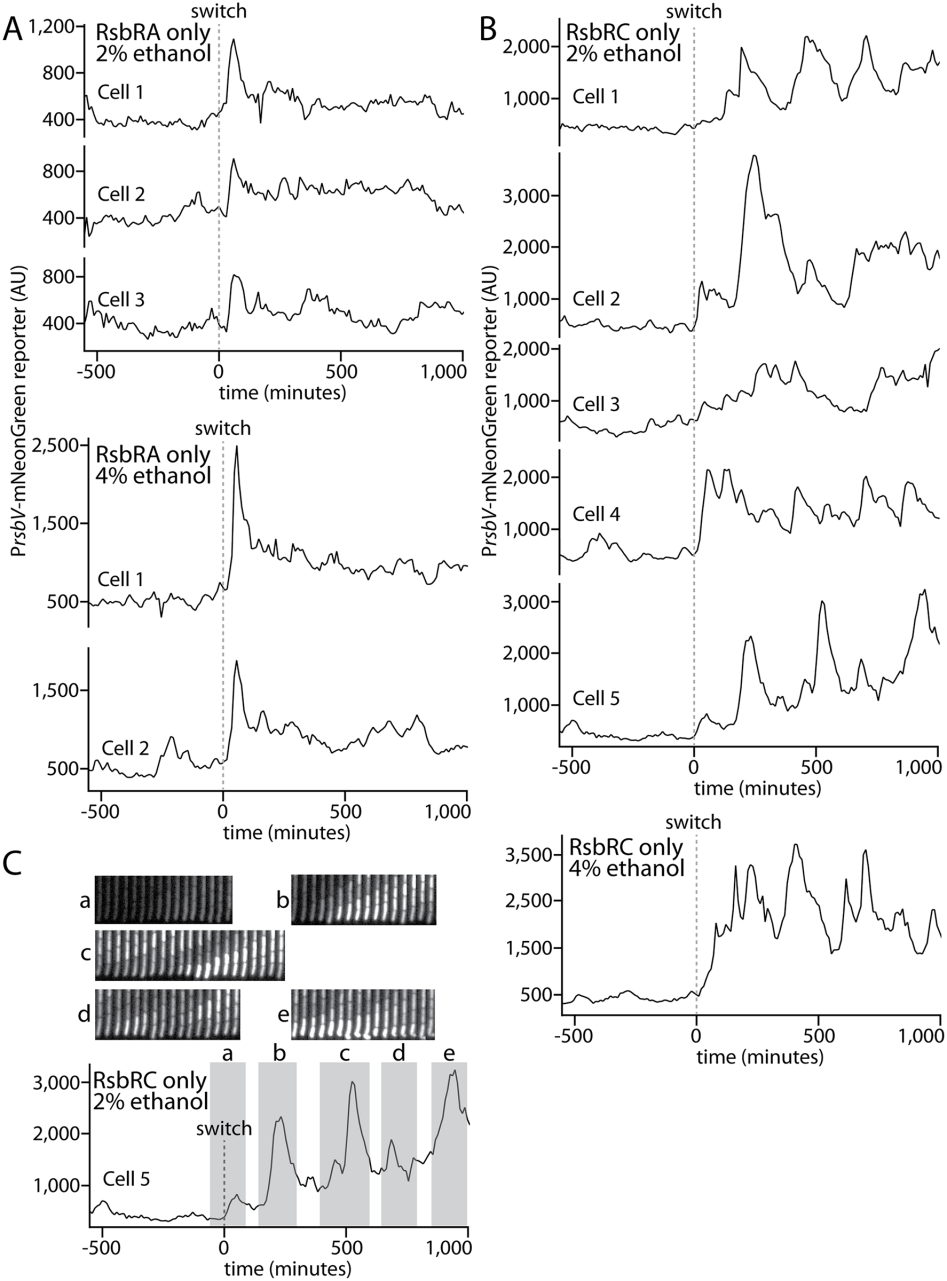
Individual cell lineages show stereotyped and stochastic response profiles in RsbRA-only and RsbRC-only strains, respectively. **A.** Intensity traces of a stress-responsive P_*rsbV*_-mNeonGreen reporter in an RsbRA-only strain (MTC1761) before and after (dashed line) the addition of ethanol at the indicated concentrations. Representative example cells are shown. **B.** Intensity traces as in **A** but with an RsbRC-only strain (MTC1765). Example cells were chosen to highlight the range of different observed responses. **C.** Annotated trace of Cell 5 from **B** with associated kymographs of the cells during response events.

Finally, we examined the responses of individual RsbRA-only and RsbRC-only cells to observe the similarities and differences among single cells in a stress-responding population. It was clear from our overlaid single-cell traces that the transient initial response spikes observed for RsbRA-only cells were synchronous across the population but heterogeneous in amplitude (Figs 4B and 5B), an observation that was borne out in traces considered individually (Fig 6A). After the initial response spike, the RsbRA-only responses varied from cell to cell within a relatively narrow range but with no discernible pattern, suggesting stochastic fluctuations in individual cells. In contrast, RsbRC-only cells showed greater variation both in time and amplitude. The progressively increasing mean response of RsbRC appears to be due to temporal heterogeneity across the population, with some cells responding early (e.g., Cells 2 and 4 in Fig 6B) and other cells not responding until later (e.g., Cells 1, 3, and 5 in Fig 6B). The sustained mean RsbRC response, as implied by our population overlays (Figs 4B and 5B), was composed not of sustained responses in single cells but rather by repeated, pulsatile activation events that varied widely in frequency and amplitude, although the amplitudes of individual events were generally greater in 4% ethanol than in 2% ethanol (Fig 6B).

We were unable to discern any patterns or characteristic frequencies in the single-cell response profiles, leading us to conclude that the pulsatile responses are governed by stochastic processes rather than oscillatory mechanisms. To test whether the response peaks were associated with cell division or other obvious morphological cues, we also visually examined cells as they underwent response events. We observed no such cues in cells exhibiting weak or strong responses; cells appeared to have normal morphology and to grow and divide normally (Fig 6C). Moreover, the response peaks often lasted for more than one cell cycle (Fig 6C), and we did not observe any obvious correlation between pulsatile responses and cell-division events (S9 Fig), although activation of the general stress response under stress generally slowed the cell growth rate (S6 Fig). Such a growth slowdown is expected because of the substantial metabolic burden of activating the general stress response.

Notably, in both RsbRA- and RsbRC-only cells, the heterogeneity across the cell population during sustained long-term responses could in principle represent a bet-hedging strategy. In this scenario, individual cells with a weaker response might be better prepared to take advantage of future decreases in stress, whereas cells with a stronger response might be poised to handle future increases in stress.

## Discussion

The use of a microfluidics-based platform to characterize the general stress response of *B*. *subtilis* cells has important advantages over earlier approaches. Unlike the use of agarose pads, the platform allows us to observe the response of cells to a stressor immediately after its application and under uniform conditions. Cells growing on agarose pads are in a potentially heterogeneous environment of progressively increasing crowding and must be exposed to a stressor before the cells are placed on pads and measurements of responses can be taken. Moreover, in contrast both to bulk (shaking liquid) culture-based experiments and to agarose pad experiments, the platform allows us to monitor the behavior of individual cells under tightly controlled conditions for long periods of time.

We used microfluidics to study the activation of σ^B^ in response to energy and environmental stress, aiming to discern general features of the bacterial stress response in the absence of extrinsic variability. To further focus on the intrinsic responses of cells, we used relatively low levels of stress that elicited a σ^B^-mediated response but did not strongly affect cell growth (S2B and S6 Figs) or survival. Our study uncovered at least three general features. First, we were able to distinguish the initial response upon stressor exposure from the long-term responses of cells that were continuously exposed to a stressor. In the case of energy stress, we observed amplitude-modulated initial responses but minimal long-term responses. With environmental stress, different ethanol concentrations produced different response patterns, as moving from 2% to 4% ethanol elicited a long-term sustained response without substantially increasing the amplitude of the initial response. Moreover, individual RsbR paralogs showed differing initial and long-term responses to identical stress conditions. For example, RsbRA-only cells had a rapid initial response but a relatively weak sustained response, whereas RsbRC-only cells were slower to initially respond but had a much stronger sustained response. Second, for environmental stress, we observed a relatively switch-like response in which the response went from barely detectable to nearly saturated over a roughly twofold increase in stressor concentration. The stressosome has been proposed as a cooperative complex [38], possibly explaining why the environmental-stress response was more switch-like than the energy-stress response. Third, we observed response heterogeneity both over time and across cell populations that was especially apparent in the pulsatile responses of single cells during long-term responses. Such heterogeneity may be especially important in a stress-response context, as some cells are (randomly) poised to take advantage of future changes in growth conditions, either for the better or for the worse.

To trigger energy stress we used the uncoupler of oxidative phosphorylation CCCP, which has been traditionally used in the field as a direct inhibitor of ATP synthesis [31, 32]. Our principal finding is that the response is amplitude-modulated with increasing levels of the uncoupler. Our results with CCCP are in contrast with the observation using mycophenolic acid as a stressor that increasing energy stress increases the frequency of responses rather than their amplitude [30]. When we tested mycophenolic acid, we saw that cells were tenfold slower to reach a half-maximal level than for CCCP and exhibited a sustained rather than transient response that provided little evidence for pulsing (S1 Fig). We note that only the sustained phase would have been visualized on agarose pads as previously used [30]. A slow σ^B^ response of cells to mycophenolic acid was also observed in bulk culture, where it was attributed to guanine nucleotide depletion [32]. Our results are in accord with the idea that mycophenolic acid provokes a different energy-stress response than CCCP because guanine nucleotide depletion, which indirectly affects ATP levels, has additional effects on cell physiology. Nonetheless, and at a minimum, our results with the microfluidic platform show that frequency modulation is not a general feature of the response to energy stress.

A longstanding question has been whether the four RsbR paralogs make distinct contributions to the environmental-stress response of *B*. *subtilis*. A few distinctions have been uncovered; for example, the blue-light sensor YtvA requires RsbRA for its light-sensing function but is negatively influenced by RsbRB [35], whereas RsbRC and RsbRD reportedly respond to nutritional stress in strains deleted for *rsbRA* and *rsbRB* [39]. However, differences among the RsbR paralogs with respect to their response profiles, even when examined individually, have been difficult to discern [22, 24, 35]. In contrast, we observed clear differences among the RsbR proteins, with each showing a different response profile to identical ethanol stress conditions. We attribute our ability to make such distinctions in large part to the long observation window and constant conditions of microfluidic devices, which permit the sustained phase of the response to be observed. In contrast, previous stress experiments in bulk culture typically tracked the response for 90 minutes or less to ensure that the cells remained in exponential phase. In retrospect, some shorter-term experiments hint at the differences we observed, for instance the sustained response of RsbRC-only cells [22]. The distinct responses of the different RsbR paralogs to the relatively low levels of ethanol stress we used in this study raise the possibility that different stressors, or more-extreme stress conditions under which the σ^B^ response becomes important for cell survival [11, 12], will reveal additional functional distinctions among the RsbR paralogs.

The close similarity between the wild-type and RsbRA-only responses, characterized by a fast but transient response (Fig 4C), suggests that the wild-type response is dominated by RsbRA. How might RsbRA dominate the overall response? Stressosomes deactivate or activate the environmental-stress response by sequestering or releasing RsbT molecules, respectively. In the stressosome structure, each sequestered RsbT protein is associated with an RsbS protein and an RsbR dimer [23] Importantly, RsbRA appears to be present at a roughly tenfold excess over RsbT [37], suggesting that there is more than enough RsbRA distributed among wild-type stressosomes to sequester all cellular RsbT. We hypothesize that RsbRA, following its initial activation by the onset of environmental stress, becomes refractory to further activation. Refractory RsbRA molecules distributed among the cellular stressosomes would then be able to recapture on their associated RsbS proteins free RsbT molecules but would be unable to re-release them. RsbT proteins freed from other RsbR paralogs would tend to accumulate on RsbS proteins associated with refractory RsbRA molecules, irrespective of the abundance or activation state of the other RsbR proteins. The sequestration of most of the cellular RsbT by refractory RsbRA molecules would give rise to the transience of the wild-type environmental-stress response and would mask the activation of the other RsbR paralogs. Therefore, only when present as the sole source of RsbR in the cell does each of the other RsbR proteins manifest its distinct response. What is the basis for such a refractory state? A refractory state of RsbRA could be induced by RsbT-mediated phosphorylation of RsbRA on its conserved C-terminal threonine residue (Fig 3A). Such phosphorylation has been suggested as a means of negative feedback under strong environmental stresses [40]. Moreover, a phosphomimetic substitution of this residue in an otherwise wild-type context markedly suppresses the environmental-stress response [17, 22], thereby resembling a refractory state.

The dominance of RsbRA in the wild-type response to ethanol argues that RsbRA may be a cellular control point in this environmental stress response; altering its levels by controlling its synthesis and/or degradation could in principle change the overall response. Activity-attenuating threonine phosphorylation of RsbRA was only detected under strong heat and ethanol stresses [40]. It is possible that in the presence of other stressors RsbRA does not become refractory, thereby permitting a different paralog to dominate the response. It will be interesting in future work to determine whether cells modulate the relative abundances and/or stabilities of the different RsbR proteins under different stress conditions to generate a particular response profile. For instance, *rsbRD* expression is stimulated by stress, and, as mentioned above, RsbT levels are approximately 10-fold lower than RsbRA levels despite being co-transcribed [37], suggesting that cells employ both transcriptional and post-transcriptional regulatory mechanisms.

In sum, the use of a microfluidic platform has proven to be an effective tool for monitoring the response to individual cells under tightly controlled conditions to energy and environmental stress. We were able to discern general stress-response features, namely that cells have distinct initial and long-term stress responses, show a relatively switch-like response to increasing stressor levels, and show response heterogeneity in time and across a population. We also conclude that energy stress is amplitude-modulated rather than frequency-modulated under the conditions of our experiments and that, in extension of earlier investigations based on bulk measurements, different RsbR proteins are capable of making decidedly different contributions to the response to environmental stress.

## Materials and methods

### Strains and growth conditions

The bacterial strains used in this study are listed in Table 1 and in the S1 Text. *B*. *subtilis* strains were routinely grown in Lennox broth (10 g/L tryptone, 5 g/L yeast extract, 5 g/L NaCl) or on Lennox agar plates fortified with 1.5% Bacto agar at 37°C. When appropriate, antibiotics (5 μg/ml chloramphenicol, 100 μg/ml spectinomycin, or 10 μg/ml tetracycline) were added to select for markers. All strains used for microfluidic analysis contained the *hag*_A233V_ point mutation to render cells immotile, thereby preventing cell loss from side channels without interfering with motility regulation [29]. Markerless deletions in *B*. *subtilis* were generated using the pMiniMAD vector (a gift of Daniel Kearns) for allelic replacement. The details of plasmid and strain construction are described in the Supplemental Text. For energy-stress experiments, cells were grown in salt-free LB medium buffered with potassium phosphate (10 g/L tryptone, 5 g/L yeast extract, 21 mM K_2_HPO_4_, and 11 mM KH_2_PO_4_) [3], as this medium reportedly enhances the response of cells to CCCP [41]. For environmental-stress experiments, standard Lennox broth was used. CCCP and mycophenolic acid were added from 100-mM stocks in DMSO, and absolute ethanol was added to the desired final concentration.

**Table 1:** Strains used in this study.

Strain or plasmid	Relevant genotype or description	Source or reference
MTC1761	3610 *hag*_*A233V*_ Δ*ytvA* Δ*rsbRB* Δ*rsbRC* Δ*rsbRD amyE*::*DG364-P*_*hyperspank*_*-mNeptune* (Cm^R^) *ywrK*::*DG1730-P*_*rsbV*_*-mNeonGreen* (Spc^R^)	This study
MTC1763	3610 *hag*_*A233V*_ Δ*ytvA* Δ*rsbRA* Δ*rsbRC* Δ*rsbRD amyE*::*DG364-P*_*hyperspank*_*-mNeptune* (Cm^R^) *ywrK*::*DG1730-P*_*rsbV*_*-mNeonGreen* (Spc^R^)	This study
MTC1765	3610 *hag*_*A233V*_ Δ*ytvA* Δ*rsbRA* Δ*rsbRB* Δ*rsbRD amyE*::*DG364-P*_*hyperspank*_*-mNeptune* (Cm^R^) *ywrK*::*DG1730-P*_*rsbV*_*-mNeonGreen* (Spc^R^)	This study
MTC1767	3610 *hag*_*A233V*_ Δ*ytvA* Δ*rsbRA* Δ*rsbRB* Δ*rsbRC amyE*::*DG364-P*_*hyperspank*_*-mNeptune* (Cm^R^) *ywrK*::*DG1730-P*_*rsbV*_*-mNeonGreen* (Spc^R^)	This study
MTC1801	3610 *hag*_*A233V*_ Δ*ytvA amyE*::*DG364-P*_*hyperspank*_*-mNeptune* (Cm^R^) *ywrK*::*DG1730-P*_*rsbV*_*-mNeonGreen* (Spc^R^)	This study
MTC1906	3610 *hag*_*A233V*_ Δ*ytvA* Δ*rsbP amyE*::*DG364-P*_*hyperspank*_*-mNeptune* (Cm^R^) *ywrK*::*DG1730-P*_*rsbV*_*-mNeonGreen* (Spc^R^)	This study
MTC1920	3610 *hag*_*A233V*_ Δ*ytvA* Δ*rsbU amyE*::*DG364-P*_*hyperspank*_*-mNeptune* (Cm^R^) *ywrK*::*DG1730-P*_*rsbV*_*-mNeonGreen* (Spc^R^)	This study
MTC1930	3610 *hag*_*A233V*_ Δ*ytvA* Δ*rsbU* Δ*rsbP amyE*::*DG364-P*_*hyperspank*_*-mNeptune* (Cm^R^) *ywrK*::*DG1730-P*_*rsbV*_*-mNeonGreen* (Spc^R^)	This study

Note: The designation delta (Δ) is used in genotypes to denote markerless, in-frame deletions of the listed genes.

### Microfluidic apparatus setup and media

The polydimethyl siloxane (PDMS) microfluidic devices were dimensionally identical to and prepared essentially as those previously described [29]. Briefly, cured PDMS devices (10:1 Sylgard 184) prepared from a silicon wafer master were punched with a 0.75-mm biopsy punch to create holes to connect the fluidics using 21-ga blunt needles. The devices were bonded to isopropyl alcohol-cleaned glass cover slips by oxygen-plasma treatment at ~200 mtorr O_2_ for 15 sec at 30 W and baked at 65°C for at least 1 hour before use. The devices were passivated with growth medium containing 1 mg/ml bovine serum albumin (BSA) before cell loading. Cells were grown in shaking culture to stationary phase (OD_600_ = ~4–5), filtered through a 5-μM filter to remove cell chains, concentrated by centrifugation at 5,000 *g* for 10 min, and loaded into the device using gel-loading tips. Cells were then spun into the side channels of the device in a custom-designed microcentrifuge adaptor at 6,000 *g* for 10 min. The fluidics were then connected to the device and run at 35 μl/min for approximately 20 min to flush out excess cells before being run at 1.5 μl/min for imaging. Imaging was not initiated until cells in the device had resumed uniform exponential growth.

The media used for fluidics always contained 1 mg/ml BSA as a passivation agent to limit cell adhesion to the device during flow. The fluidics were fed by 20-ml syringes in 6-channel syringe pumps (New Era Pump Systems, Farmingdale NY) that were connected by 21-ga blunt needles to Tygon flexible tubing with an inner diameter (ID) of 0.02". To permit medium switches, 2 banks of syringes were used, one for the one for the pre-stress phase containing plain medium, the other for the stress phase containing the stressor. Each pair of syringes (minus and plus stressor) was joined with a polypropylene 1.6-mm ID Y-connector with 200-series barbs; 2-cm lengths of flexible silicone tubing (0.04" ID, 0.085" outer diameter) were used to connect the Tygon lines to the 2 input branches Y-connector. The lengths of silicone tubing facilitated the placement of small binder clips to one or the other branch to make pinch valves. A 1-cm length of silicone tubing was used to connect a 10-cm length of Tygon tubing to the output of the Y connector. The output Tygon tubing was directly connected to a bent 21-ga blunt needle that was then connected to the PDMS device, and a similarly constructed needle-tube combination was used to carry the outflow of the device to a waste beaker.

In initial tests, we noticed that a higher concentration of CCCP was necessary to elicit a response under microfluidic conditions than under flask-grown conditions. We attribute this difference to the continuous flow and "open" nature of the microfluidic system, which likely attenuates nutritional stress because cells are continuously supplied with nutrients.

### Medium switching

Experiments were always initiated in stressor-free medium, and this initial growth phase typically lasted approximately 10–12 h before the switch. In the initial phase, pinch valves were closed on the stressor-containing branch of the fluidics, and the corresponding syringe pump was paused. At the switch, the syringe pump with stressor-free medium was paused, the binder clips were carefully moved to the stressor-free branch of the Y-connectors, and the other (stressor-containing) syringe pump was activated (at 1.5 μl/min). Initial tests with marker beads and dyes indicated that the second medium took approximately 50 minutes to reach the cells in the device. The switch apparatus was housed within a temperature-controlled microscope enclosure during imaging (see below).

### Automated imaging

Imaging was performed with a Nikon Eclipse Ti inverted microscope equipped with an Orca R2 (Hamamatsu) camera, a 60X Plan Apo oil objective (NA 1.4, Nikon), an automated stage (Ludl), a Lumencor SOLA fluorescent illumination system, and a custom-designed temperature-controlled Plexiglas enclosure in which the temperature was maintained at approximately 37°C during imaging. Image acquisition was performed using MATLAB scripts interfacing with μManager, as previously described [29]. Semrock filter cubes for GFP (GFP1828A) and mKate2 (mCherry-B) were used to image mNeonGreen and mNeptune, respectively. mNeonGreen (used for σ^B^ reporters) was imaged with 2x2 pixel binning at approximately 20% illumination power with 200-ms exposures, and mNeptune (used for cell segmentation) was imaged with 1x1 binning at approximately 24% power with 400-ms exposures). Images were captured at 10-minute intervals.

### Segmentation, lineage tracking and curation

Automated cell segmentation and lineage tracking was performed as previously described [29] using a constitutively expressed cytoplasmic mNeptune reporter. The average mNeonGreen average intensity in mother cells was used to generate σ^B^ reporter traces. After lineage tracking, lineages were filtered to retain only lineages that were tracked for >150 continuous frames. Because spontaneous cell death events and other anomalies (e.g., overcrowding of side channels) were associated with spurious peaks in reporter intensity, the full filtered set of lineages was then manually curated to remove spurious events. The average traces for the full and curated lineage sets showed close concordance, as shown in S2–S5 Figs, implying that the curation process did not bias the overall observed trends. In all other figures, curated lineage sets were used to plot average traces and overlaid single-cell traces.

### Cell division time analysis

As part of the automated cell-segmentation process, cell lengths and division times are calculated. For display, we averaged the observed division times over a 10-frame sliding window (an example of a scatter plot and the corresponding averaged trace is shown in S11 Fig). In all cases, the data from the full set and the curated set were virtually indistinguishable (S11 Fig), so only full-set data are shown for simplicity.

## Supporting information

S1 Text Detailed modes of strain construction, complete strain and plasmid tables, and primer sequences. (PDF)

S1 Fig Response of wild-type cells to 60 μg/ml mycophenolic acid (MPA) as an energy stressor. **A.** Single-cell intensity traces of a stress-responsive P_*rsbV*_-mNeonGreen reporter before and after (dashed line) the addition of MPA. **B.** Two example traces from the ensemble of traces shown in Panel A are shown. **C.** The average trace from the curated set of cell-lineages shown in Panel A is shown along with the standard deviation (gray envelope surrounding the mean trace). **D.** Comparison of the average response profile of the full set of lineages from the experiment (black) and the curated set from the same experiment from which lineages displaying cell death, tracking errors, or other artifacts were removed.
(TIF)

S2 Fig Average response traces and cell division times in cells experiencing energy stress. **A.** Comparisons of the average response profiles from the full set of lineages from a particular experiment (black) and the curated set from the same experiment (gray) from which lineages displaying cell death, tracking errors, or other artifacts were removed. The stress conditions, beginning at the dashed line, are indicated in each plot. **B.** Comparisons of averaged division times in cell populations experiencing the indicated stress conditions (beginning at the dashed line).
(TIF)

S3 Fig Average response traces in wild-type cells experiencing different levels of ethanol stress. Comparisons of the average response profiles from the full set of lineages from a particular experiment (black) and the curated set from the same experiment (gray) from which lineages displaying cell death, tracking errors, or other artifacts were removed. The stress conditions, beginning at the dashed line, are indicated in each plot.
(TIF)

S4 Fig Average response traces in single-RsbR cells experiencing 2% ethanol stress. Comparisons of the average response profiles from the full set of lineages from a particular experiment (black) and the curated set from the same experiment (gray) from which lineages displaying cell death, tracking errors, or other artifacts were removed. The strain and stress conditions, beginning at the dashed line, are indicated in each plot.
(TIF)

S5 Fig Average response traces of RsbRA- or RsbRC-only cells experiencing different levels of ethanol stress. **A.** Comparisons of the average response profiles of RsbRA-only cells from the full set of lineages from a particular experiment (black) and the curated set from the same experiment (gray) from which lineages displaying cell death, tracking errors, or other artifacts were removed. The strain and stress conditions, beginning at the dashed line, are indicated in each plot. **B.** Distributions of signal increases in single RsbRA-only cells from before stress exposure to their maximal peak values. All differences were statistically significant (*p* < 6.8 x 10^−7^). **C.** Comparisons of the average response profiles of RsbRC-only cells from the full set of lineages from a particular experiment (black) and the curated set from the same experiment (gray) from which lineages displaying cell death, tracking errors, or other artifacts were removed. The strain and stress conditions, beginning at the dashed line, are indicated in each plot.
(TIF)

S6 Fig Average division-time traces of wild-type and single-RsbR strains at different ethanol stress levels. The left panels show overlaid division-time traces at different levels of ethanol stress for the listed strains. The right panels show overlaid division-time traces for different strains as listed in each plot. All traces are averages of a 10-frame sliding window, and the average of the full (uncurated) set of lineages is shown in each case.
(TIF)

S7 Fig Average response traces showing standard deviations. Average traces of the curated cell-lineage sets from the listed strains at the listed ethanol concentrations are shown together with the standard deviation (gray envelope surrounding the mean trace).
(TIF)

S8 Fig Coefficient-of-variance traces of different strains under different levels of ethanol stress. The left panels show overlaid traces at different levels of ethanol stress for the listed strains. The right panels show overlaid traces from different strains at the listed ethanol concentrations.
(TIF)

S9 Fig Single-cell stress-response profiles with corresponding cell-length traces. The strain and stress condition for each plot are listed; the example cell numbers correspond to those shown in Fig 5. The top of each plot shows the P_*rsbV*_-mNeonGreen reporter intensity, while the bottom of each plot shows cell length, as automatically calculated from constitutive-fluorophore (red, mNeptune) images. For ease of visualization, alternating gray and white bars are placed between consecutive cell-division events, inferred from steep downward slopes in the cell-length traces.
(TIF)

S10 Fig Response profiles of strains lacking RsbP and RsbU. **Left panels**, response traces of a Δ*rsbPU* strain (MTC1930) expected to be unresponsive to stress. This experiment was conducted to observe the frequency and magnitude of σ^B^ activation events in the absence of both upstream stress-signaling pathways (environmental and energy stress) in unstressed conditions. **Right panels**, the response to 2% ethanol (dashed line) in Δ*rsbU* (MTC1920) cells otherwise wild-type for environmental stress (i.e., containing all four RsbR paralogs). The top graphs show overlaid single-cell traces, while the bottom graphs show mean traces with a standard-deviation envelope (gray).
(TIF)

S11 Fig Example plots of cell division times in the microfluidic device. **Top panel**, a plot of individual-cell division times (dots) along the course of a representative experiment. The time on the Y-axis indicates the time since the last division; the resolution of the method is 10 minutes due to the 10-minute imaging interval used in the experiment. Cell-division events were automatically computed from a constitutive marker (P_*hyperspank*_-mNeptune) visible in the red fluorescence channel. The blue trace shows the population division time averaged over a 10-frame sliding window. **Bottom panel**, a comparison of the average division time plots as computed from the full set of lineages (blue) or the curated set (red) from which lineages displaying cell death, tracking errors, or other artifacts were removed. The traces were indistiguishable in all cases, so we show only the full-set traces in subsequent figures.
(TIF)

S1 Movie Response of wild-type cells to the onset of 40 μM CCCP. Representative MTC1801 cell lineages are shown, with the mother cells oriented toward the bottom of the frame and the feeding/waste channel toward the top of the frame. The images were captured in the GFP channel to visualize the P_*rsbV*_-mNeonGreen reporter. Time is shown in hours and minutes, and the introduction of CCCP corresponds with the appearance of the label.
(AVI)

S2 Movie Response of wild-type cells to the onset of 25 μM CCCP. Representative MTC1801 cell lineages are shown, with the mother cells oriented toward the bottom of the frame and the feeding/waste channel toward the top of the frame. The images were captured in the GFP channel to visualize the P_*rsbV*_-mNeonGreen reporter. Time is shown in hours and minutes, and the introduction of CCCP corresponds with the appearance of the label.
(AVI)

S3 Movie Response of wild-type cells to the onset of 55 μM CCCP. Representative MTC1801 cell lineages are shown, with the mother cells oriented toward the bottom of the frame and the feeding/waste channel toward the top of the frame. The images were captured in the GFP channel to visualize the P_*rsbV*_-mNeonGreen reporter. Time is shown in hours and minutes, and the introduction of CCCP corresponds with the appearance of the label.
(AVI)

S4 Movie Response of wild-type cells to DMSO vehicle. Representative MTC1801 cell lineages are shown, with the mother cells oriented toward the bottom of the frame and the feeding/waste channel toward the top of the frame. The images were captured in the GFP channel to visualize the P_*rsbV*_-mNeonGreen reporter. Time is shown in hours and minutes, and the introduction of DMSO (0.055%, corresponding to the DMSO concentration added to yield 55 μM CCCP) corresponds with the appearance of the label.
(AVI)

S5 Movie Response of Δ*rsbP* cells to the onset of 55 μM CCCP. Representative MTC1906 cell lineages are shown, with the mother cells oriented toward the bottom of the frame and the feeding/waste channel toward the top of the frame. The images were captured in the GFP channel to visualize the P_*rsbV*_-mNeonGreen reporter. Time is shown in hours and minutes, and the introduction of CCCP corresponds with the appearance of the label.
(AVI)

S6 Movie Response of wild-type cells to the onset of 60 μg/ml mycophenolic acid. Representative MTC1801 cell lineages are shown, with the mother cells oriented toward the bottom of the frame and the feeding/waste channel toward the top of the frame. The images were captured in the GFP channel to visualize the P_*rsbV*_-mNeonGreen reporter. Time is shown in hours and minutes, and the introduction of mycophenolic acid (MPA) corresponds with the appearance of the label.
(AVI)

S7 Movie Response of wild-type cells to the onset of 1% ethanol. Representative MTC1801 cell lineages are shown, with the mother cells oriented toward the bottom of the frame and the feeding/waste channel toward the top of the frame. The images were captured in the GFP channel to visualize the P_*rsbV*_-mNeonGreen reporter. Time is shown in hours and minutes, and the introduction of ethanol corresponds with the appearance of the label.
(AVI)

S8 Movie Response of wild-type cells to the onset of 2% ethanol. Representative MTC1801 cell lineages are shown, with the mother cells oriented toward the bottom of the frame and the feeding/waste channel toward the top of the frame. The images were captured in the GFP channel to visualize the P_*rsbV*_-mNeonGreen reporter. Time is shown in hours and minutes, and the introduction of ethanol corresponds with the appearance of the label.
(AVI)

S9 Movie Response of Δ*rsbU* cells to the onset of 2% ethanol. Representative MTC1920 cell lineages are shown, with the mother cells oriented toward the bottom of the frame and the feeding/waste channel toward the top of the frame. The images were captured in the GFP channel to visualize the P_*rsbV*_-mNeonGreen reporter. Time is shown in hours and minutes, and the introduction of ethanol corresponds with the appearance of the label.
(AVI)

S10 Movie Response of wild-type cells to the onset of 4% ethanol. Representative MTC1801 cell lineages are shown, with the mother cells oriented toward the bottom of the frame and the feeding/waste channel toward the top of the frame. The images were captured in the GFP channel to visualize the P_*rsbV*_-mNeonGreen reporter. Time is shown in hours and minutes, and the introduction of ethanol corresponds with the appearance of the label.
(AVI)

S11 Movie Response of RsbRA-only cells to the onset of 2% ethanol. Representative MTC1761 cell lineages are shown, with the mother cells oriented toward the bottom of the frame and the feeding/waste channel toward the top of the frame. The images were captured in the GFP channel to visualize the P_*rsbV*_-mNeonGreen reporter. Time is shown in hours and minutes, and the introduction of ethanol corresponds with the appearance of the label.
(AVI)

S12 Movie Response of RsbRC-only cells to the onset of 2% ethanol. Representative MTC1765 cell lineages are shown, with the mother cells oriented toward the bottom of the frame and the feeding/waste channel toward the top of the frame. The images were captured in the GFP channel to visualize the P_*rsbV*_-mNeonGreen reporter. Time is shown in hours and minutes, and the introduction of ethanol corresponds with the appearance of the label.
(AVI)

S13 Movie Response of RsbRB-only cells to the onset of 2% ethanol. Representative MTC1763 cell lineages are shown, with the mother cells oriented toward the bottom of the frame and the feeding/waste channel toward the top of the frame. The images were captured in the GFP channel to visualize the P_*rsbV*_-mNeonGreen reporter. Time is shown in hours and minutes, and the introduction of ethanol corresponds with the appearance of the label.
(AVI)

S14 Movie Response of RsbRD-only cells to the onset of 2% ethanol. Representative MTC1767 cell lineages are shown, with the mother cells oriented toward the bottom of the frame and the feeding/waste channel toward the top of the frame. The images were captured in the GFP channel to visualize the P_*rsbV*_-mNeonGreen reporter. Time is shown in hours and minutes, and the introduction of ethanol corresponds with the appearance of the label.
(AVI)

S15 Movie Response of RsbRA-only cells to the onset of 1% ethanol. Representative MTC1761 cell lineages are shown, with the mother cells oriented toward the bottom of the frame and the feeding/waste channel toward the top of the frame. The images were captured in the GFP channel to visualize the P_*rsbV*_-mNeonGreen reporter. Time is shown in hours and minutes, and the introduction of ethanol corresponds with the appearance of the label.
(AVI)

S16 Movie Response of RsbRC-only cells to the onset of 1% ethanol. Representative MTC1765 cell lineages are shown, with the mother cells oriented toward the bottom of the frame and the feeding/waste channel toward the top of the frame. The images were captured in the GFP channel to visualize the P_*rsbV*_-mNeonGreen reporter. Time is shown in hours and minutes, and the introduction of ethanol corresponds with the appearance of the label.
(AVI)

S17 Movie Response of RsbRC-only cells to the onset of 4% ethanol. Representative MTC1765 cell lineages are shown, with the mother cells oriented toward the bottom of the frame and the feeding/waste channel toward the top of the frame. The images were captured in the GFP channel to visualize the P_*rsbV*_-mNeonGreen reporter. Time is shown in hours and minutes, and the introduction of ethanol corresponds with the appearance of the label.
(AVI)

S18 Movie Response of RsbRA-only cells to the onset of 4% ethanol. Representative MTC1761 cell lineages are shown, with the mother cells oriented toward the bottom of the frame and the feeding/waste channel toward the top of the frame. The images were captured in the GFP channel to visualize the P_*rsbV*_-mNeonGreen reporter. Time is shown in hours and minutes, and the introduction of ethanol corresponds with the appearance of the label.
(AVI)
