# Supplementary material for: Use of a microfluidic platform to uncover basic features of energy and environmental stress responses in individual cells of Bacillus subtilis
Source: PLoS Genet. 2017 Jul 20;13(7):e1006901. doi: 10.1371/journal.pgen.1006901 (PMC5542698; doi:10.1371/journal.pgen.1006901)
Supplement: S1 Text — (PDF) [file pgen.1006901.s001.pdf]

Detailed Modes of Strain and Plasmid Construction

The markerless deletions in the strains below were added in succession using the pminiMAD vector (gift of Daniel Kearns). For each deletion step, a pminiMAD vector containing the desired deletion was directly transformed into PY79 via competence {Wilson, 1968 #1148} and selected on MLS (0.5 μg/ml erythromycin and 2.5 μg/ml lincomycin). A phage SPP1 lysate was prepared from that intermediate strain, and the recipient strain was phage-transduced with the PY79 strain containing the desired chromosomally integrated miniMAD vector and again selected on MLS. Five to 10 transductants were then inoculated into liquid LB and kept in exponential phase at approximately 25°C for several hours to permit plasmid excision before being repeatedly diluted and grown in liquid LB at 37°C (restrictive for plasmid replication) to promote loss of excised plasmid. The cells were then plated, and single colonies were screened for the deletion by colony PCR, restreaked for single colonies, patched on plain LB and LB/MLS plates to verify plasmid loss, restreaked, verified by PCR and stored at -80°C.

MTC1761

Wild-type NCIB3610 (RL2912) was deleted for *ytvA* to produce MTC1672 (3610 Δ*ytvA*). MTC1672 was deleted for *rsbRC* to produce MTC1685 (3610 Δ*ytvA* Δ*rsbRC*). MTC1685 was deleted for *rsbRD* to produce MTC1689 (3610 Δ*ytvA* Δ*rsbRC* Δ*rsbRD*). MTC1689 was deleted for *rsbRB* to produce MTC1697 (3610 Δ*ytvA* Δ*rsbRB* Δ*rsbRC* Δ*rsbRD*). MTC1697 was phage-transduced with a lysate from TMN688 (3610 hag_{A233V} $phleo^R$) and selected on 20 μg/ml zeocin to produce MTC1707 (3610 Δ*ytvA* Δ*rsbRB* Δ*rsbRC* Δ*rsbRD* hag_{A233V}). MTC1707 was transduced with a lysate from MTC1741 (PY79 *ywrK*::*DG1730*-P_{rsbV}-*mNeonGreen*) and selected on spectinomycin (100 μg/ml) to produce MTC1749 (3610 Δ*ytvA* Δ*rsbRB* Δ*rsbRC* Δ*rsbRD* hag_{A233V} *ywrK*::*DG1730*-P_{rsbV}-*mNeonGreen*). Finally, MTC1749 was transduced with a lysate from JRR227 (PY79 *amyE*::*DG364*-$P_{hyperspank}$-*mNeptune*) and selected on chloramphenicol (5 μg/ml) to produce MTC1761.

MTC1763

Constructed as MTC1761, except that MTC1689 was deleted for *rsbRA* to produce MTC1695 (3610 Δ*ytvA* Δ*rsbRA* Δ*rsbRC* Δ*rsbRD*). MTC1695 was phage-transduced with hag_{A233V} to produce MTC1705, which was transduced with lysate from MTC1741 to produce MTC1750, which was transduced with lysate from JRR227 to produce MTC1763.

MTC1765

Constructed as MTC1761, except that MTC1672 was deleted for *rsbRA* to produce MTC1683 (3610 Δ*ytvA* Δ*rsbRA*). MTC1683 was deleted for *rsbRB* to produce MTC1687 (3610 Δ*ytvA* Δ*rsbRA* Δ*rsbRB*). MTC1687 was deleted for *rsbRD* to produce MTC1693 (3610 Δ*ytvA* Δ*rsbRA* Δ*rsbRB* Δ*rsbRD*). MTC1693 was phage-transduced with hag_{A233V} to produce MTC1703, which

was transduced with lysate from MTC1741 to produce MTC1751, which was transduced with lysate from JRR227 to produce MTC1765.

MTC1767

Constructed as MTC1765, except that MTC1687 (3610 Δ*ytvA* Δ*rsbRA* Δ*rsbRB*) was deleted for *rsbRC* to produce MTC1691 (3610 Δ*ytvA* Δ*rsbRA* Δ*rsbRB* Δ*rsbRC*). MTC1691 was phage-transduced with hag_{A233V} to produce MTC1701, which was transduced with lysate from MTC1741 to produce MTC1752, which was transduced with lysate from JRR227 to produce MTC1767.

MTC1801

Constructed as MTC1761, except that MTC1672 (3610 Δ*ytvA*) was phage-transduced with hag_{A233V} to produce MTC1779, which was transduced with lysate from JRR227 to produce MTC1793, which was transduced with lysate from MTC1741 to produce MTC1801.

MTC1906

Constructed as MTC1801, except that MTC1779 (3610 Δ*ytvA* hag_{A233V}) was deleted for *rsbP* to produce MTC1898 (3610 Δ*ytvA* Δ*rsbP* hag_{A233V}), which was transduced with lysate from JRR227 to produce MTC1902, which was transduced with lysate from MTC1741 to produce MTC1906.

MTC1920

Constructed as MTC1801, except that MTC1779 (3610 Δ*ytvA* hag_{A233V}) was deleted for *rsbU* to produce MTC1912 (3610 Δ*ytvA* Δ*rsbU* hag_{A233V}), which was transduced with lysate from JRR227 to produce MTC1916, which was transduced with lysate from MTC1741 to produce MTC1920.

MTC1930

Constructed as MTC1920, except that MTC1912 (3610 Δ*ytvA* Δ*rsbU* hag_{A233V}) was deleted for *rsbP* to produce MTC1926 (3610 Δ*ytvA* Δ*rsbP* Δ*rsbU* hag_{A233V}), which was transduced with lysate from JRR227 to produce MTC1928, which was transduced with lysate from MTC1741 to produce MTC1930.

Plasmid construction methods

pDG364-$P_{hyperspank}$-mNeptune

Plasmid pDG364 was linearized with HindIII. The *hyperspank* promoter was amplified from pDR111 (laboratory stock) with primers JRR162 and JRR163, and mNeptune was amplified

from template DNA provided by Ethan Garner with primers JRR164 and JRR165. The two amplicons were gel purified and isothermally assembled{Gibson, 2009 #989} together with the linearized pDG364. The resulting plasmid was propagated in *E. coli* and confirmed by sequencing.

pDG1730-P_{rsbV}-mNeonGreen

Plasmid pDG1730 was linearized with EcoRI and alkaline phosphatase-treated. The promoter region of *rsbV* was PCR-amplified from *B. subtilis* NCIB3610 genomic DNA with primers 727 and 728, and the mNeonGreen coding sequence was amplified from template DNA kindly provided by Ethan Garner using primers 729 and JRR189. These two amplicons were gel purified and PCR-stitched together via self priming and primers 727 and JRR189 before being gel-purified and isothermally assembled {Gibson, 2009 #989} into the linearized pDG1730. The resulting plasmid was propagated in *E. coli* and confirmed by sequencing.

pminiMAD-ΔrsbRA

Plasmid pminiMAD was linearized with EcoRI and HindIII. The 5' and 3' flanking regions (500-600 nt) of *rsbRA* were amplified with primers 689/690 and 691/692, respectively, gel purified, PCR-stitched together using primers 689/692, again gel-purified, and isothermally assembled {Gibson, 2009 #989} with the linearized pminiMAD. The resulting plasmid was propagated in *E. coli* and confirmed by sequencing.

pminiMAD-ΔrsbRB

Plasmid pminiMAD was linearized with EcoRI and HindIII. The 5' and 3' flanking regions (500-600 nt) of *rsbRB* were amplified with primers 693/694 and 695/696, respectively, gel purified, PCR-stitched together using primers 693/696, again gel-purified, and isothermally assembled {Gibson, 2009 #989} with the linearized pminiMAD. The resulting plasmid was propagated in *E. coli* and confirmed by sequencing.

pminiMAD-ΔrsbRC

Plasmid pminiMAD was linearized with EcoRI and HindIII. The 5' and 3' flanking regions (500-600 nt) of *rsbRC* were amplified with primers 697/698 and 699/700, respectively, gel purified, PCR-stitched together using primers 697/700, again gel-purified, and isothermally assembled {Gibson, 2009 #989} with the linearized pminiMAD. The resulting plasmid was propagated in *E. coli* and confirmed by sequencing.

pminiMAD-ΔrsbRD

Plasmid pminiMAD was linearized with EcoRI and HindIII. The 5' and 3' flanking regions (500-600 nt) of *rsbRD* were amplified with primers 701/702 and 703/704, respectively, gel purified,

PCR-stitched together using primers 701/704, again gel-purified, and isothermally assembled {Gibson, 2009 #989} with the linearized pminiMAD. The resulting plasmid was propagated in *E. coli* and confirmed by sequencing.

pminiMAD-ΔrsbP

Plasmid pminiMAD was linearized with EcoRI and HindIII. The 5' and 3' flanking regions (500-600 nt) of *rsbP* were amplified with primers 756/757 and 758/759, respectively, gel purified, PCR-stitched together using primers 757/759, again gel-purified, and isothermally assembled {Gibson, 2009 #989} with the linearized pminiMAD. The resulting plasmid was propagated in *E. coli* and confirmed by sequencing.

pminiMAD-ΔrsbU

Plasmid pminiMAD was linearized with EcoRI and HindIII. The 5' and 3' flanking regions (500-600 nt) of *rsbU* were amplified with primers 752/753 and 754/755, respectively, gel purified, PCR-stitched together using primers 753/755, again gel-purified, and isothermally assembled {Gibson, 2009 #989} with the linearized pminiMAD. The resulting plasmid was propagated in *E. coli* and confirmed by sequencing.

pminiMAD-ΔytvA

Plasmid pminiMAD was linearized with EcoRI and HindIII. The 5' and 3' flanking regions (500-600 nt) of *ytvA* were amplified with primers 705/706 and 707/708, respectively, gel purified, PCR-stitched together using primers 705/708, again gel-purified, and isothermally assembled {Gibson, 2009 #989} with the linearized pminiMAD. The resulting plasmid was propagated in *E. coli* and confirmed by sequencing.

Table S1. Intermediate strains, plasmids, and primers

**Strain or plasmid**	**Relevant genotype or description**	**Source or reference**
***B. subtilis* strains**		
JRR227	PY79 *amyE::DG364-*$P_{hyperspank}$*-mNeptune* (Cm^R)	This study
MTC1672	3610 Δ*ytvA*	This study
MTC1683	3610 Δ*ytvA* Δ*rsbRA*	This study
MTC1685	3610 Δ*ytvA* Δ*rsbRC*	This study
MTC1687	3610 Δ*ytvA* Δ*rsbRA* Δ*rsbRB*	This study

MTC1689	3610 Δ*ytvA* Δ*rsbRC* Δ*rsbRD*	This study
MTC1691	3610 Δ*ytvA* Δ*rsbRA* Δ*rsbRB* Δ*rsbRC*	This study
MTC1693	3610 Δ*ytvA* Δ*rsbRA* Δ*rsbRB* Δ*rsbRD*	This study
MTC1695	3610 Δ*ytvA* Δ*rsbRA* Δ*rsbRC* Δ*rsbRD*	This study
MTC1697	3610 Δ*ytvA* Δ*rsbRB* Δ*rsbRC* Δ*rsbRD*	This study
MTC1701	3610 Δ*ytvA* Δ*rsbRA* Δ*rsbRB* Δ*rsbRC* hag_{A233V} ($Phleo^R$)	This study
MTC1703	3610 Δ*ytvA* Δ*rsbRA* Δ*rsbRB* Δ*rsbRD* hag_{A233V} ($Phleo^R$)	This study
MTC1705	3610 Δ*ytvA* Δ*rsbRA* Δ*rsbRC* Δ*rsbRD* hag_{A233V} ($Phleo^R$)	This study
MTC1707	3610 Δ*ytvA* Δ*rsbRB* Δ*rsbRC* Δ*rsbRD* hag_{A233V} ($Phleo^R$)	This study
MTC1741	PY79 *ywrK*::*DG1730*-P_{rsbV}-*mNeonGreen* (Spc^R)	This study
MTC1749	3610 Δ*ytvA* Δ*rsbRB* Δ*rsbRC* Δ*rsbRD* hag_{A233V} ($Phleo^R$) *ywrK*::*DG1730*-P_{rsbV}-*mNeonGreen* (Spc^R)	This study
MTC1750	3610 Δ*ytvA* Δ*rsbRA* Δ*rsbRC* Δ*rsbRD* hag_{A233V} ($Phleo^R$) *ywrK*::*DG1730*-P_{rsbV}-*mNeonGreen* (Spc^R)	This study
MTC1751	3610 Δ*ytvA* Δ*rsbRA* Δ*rsbRB* Δ*rsbRD* hag_{A233V} ($Phleo^R$) *ywrK*::*DG1730*-P_{rsbV}-*mNeonGreen* (Spc^R)	This study
MTC1752	3610 Δ*ytvA* Δ*rsbRA* Δ*rsbRB* Δ*rsbRC* hag_{A233V} ($Phleo^R$) *ywrK*::*DG1730*-P_{rsbV}-*mNeonGreen* (Spc^R)	This study
MTC1779	3610 Δ*ytvA* hag_{A233V} ($Phleo^R$)	This study
MTC1793	3610 Δ*ytvA* hag_{A233V} ($Phleo^R$) *amyE*::*DG364*-$P_{hyperspank}$-*mNeptune* (Cm^R)	This study
MTC1898	3610 Δ*ytvA* Δ*rsbP* hag_{A233V} ($Phleo^R$)	This study
MTC1902	3610 Δ*ytvA* Δ*rsbP* hag_{A233V} ($Phleo^R$) *amyE*::*DG364*-$P_{hyperspank}$-*mNeptune* (Cm^R)	This study
MTC1912	3610 Δ*ytvA* Δ*rsbU* hag_{A233V} ($Phleo^R$)	This study
MTC1916	3610 Δ*ytvA* Δ*rsbU* hag_{A233V} ($Phleo^R$) *amyE*::*DG364*-$P_{hyperspank}$-*mNeptune* (Cm^R)	This study

MTC1926	3610 Δ*ytvA* Δ*rsbP* Δ*rsbU* hag_{A233V} ($Phleo^R$)	This study
MTC1928	3610 Δ*ytvA* Δ*rsbP* Δ*rsbU* hag_{A233V} ($Phleo^R$) *amyE*::*DG364*-$P_{hyperspank}$-*mNeptune* (Cm^R)	This study
RL2912	*B. subtilis* 3610	Laboratory stock
TMN688	3610 hag_{A233V} ($Phleo^R$)	{Norman, 2013 #1013}
**Plasmids**		
pDG364	*amyE* integrating plasmid (Cm^R)	Laboratory stock
pDG364-$P_{hyperspank}$-mNeptune	*amyE* integrating plasmid (Cm^R) carrying constitutively expressed red fluorescent protein mNeptune	This study
pDG1730	*amyE* integrating plasmid (Spc^R)	
pDG1730-P_{rsbV}-mNeonGreen	*amyE* integrating plasmid (Spc^R) carrying P_{rsbV} (σ^B) transcriptional reporter	This study
pminiMAD	Suicide plasmid for *B. subtilis* markerless allelic replacement	Gift of Daniel Kearns
pminiMAD-ΔrsbRA	pminiMAD-based markerless deletion plasmid for *rsbRA*	This study
pminiMAD-ΔrsbRB	pminiMAD-based markerless deletion plasmid for *rsbRB*	This study
pminiMAD-ΔrsbRC	pminiMAD-based markerless deletion plasmid for *rsbRC*	This study
pminiMAD-ΔrsbRD	pminiMAD-based markerless deletion plasmid for *rsbRD*	This study
pminiMA	pminiMAD-based markerless deletion plasmid for *rsbP*	This study

D-ΔrsbP		
pminiMAD-ΔrsbU	pminiMAD-based markerless deletion plasmid for *rsbU*	This study
pminiMAD-ΔytvA	pminiMAD-based markerless deletion plasmid for *ytvA*	This study
**Primer name**	**Sequence (5'-3')**	
JRR162	GATCCTAGAAGCTTATCGAACTCGAGGGTAAATGTGAGCACTC	Russell et al., 2017 (submitted)
JRR163	TCCTCGCCTTTACTTACCATGCTCATTCCTCCTAATTGTTATCCGC	Russell et al., 2017 (submitted)
JRR164	AACAATTAGGAGGAATGAGCATGGTAAGTAAAGGCGAGGAGC	Russell et al., 2017 (submitted)
JRR165	TAAGCTGTCAAACATGAGAATTACTTGTAAAGTTCGTCCATTCCATTC	Russell et al., 2017 (submitted)
JRR189	ATTCGCCAGGGCTGCAGGAATTACTTATAGAGTTCATCCATACCCATC	Russell et al., 2017 (submitted)
689	AACAGCTATGACCATGATTACGCCAAGCTTCGGCTATATGGAAATGGCG	This study
690	ATGTCTCAAAATGCAAATTCGCTTACCTCCC	This study
691	GTAAGCGAATTTGCATTTTGAGACATCCGAAAATCC	This study
692	CGTTGTAAAACGACGGCCAGTGAATTCTTCCTCTGTCTGCGACCTG	This study

693	AACAGCTATGACCATGATTACGCCAAGCTTTCGCCGCCAAGAACCTTC	This study
694	AATCCCCAGCGACACTGCTCCTTTCCCC	This study
695	GGAGCAGTGTCGCTGGGGATTGACTTTTCGA	This study
696	CGTTGTAAAACGACGGCCAGTGAATTCTGTCGGCATCTCTCTCGGG	This study
697	AACAGCTATGACCATGATTACGCCAAGCTTGGCAGCCATGAATTTTGCG	This study
698	CAAGGAAATACCGATTGATCACCTCTTTTAAATGTG	This study
699	GGTGATCAATCGGTATTTCCTTGAGATCCACTC	This study
700	CGTTGTAAAACGACGGCCAGTGAATTCCAAGAGCTCATCAACGCTTGC	This study
701	AACAGCTATGACCATGATTACGCCAAGCTTATATCCAAGCTGCACGTC	This study
702	GGCAAAGGCATCTCTTAATGAGTTACCTCCTG	This study
703	AACTCATTAAGAGATGCCTTTGCCATGTTTATC	This study
704	CGTTGTAAAACGACGGCCAGTGAATTCGTGCTGTTTTCCATGCTGAC	This study
705	AACAGCTATGACCATGATTACGCCAAGCTTGCCGTCATGCCGATAAACC	This study
706	GAGCGGGATCATAAATCCCCCTTAGGCCG	This study
707	GGGGATTTATGATCCCGCTCACCCAGC	This study
708	CGTTGTAAAACGACGGCCAGTGAATTCTCCGATGTTTCTCGCTGCG	This study
727	GATCCTAGAAGCTTATCGAAAACAATTCGATCAGCATCTGGAAAAG	This study
728	TCCTCTCCTTTCGAAACCATCAAATTTTCCTTCAAATCACTAGTTGC	This study
729	GTGATTTGAAGGAAAATTTGATGGTTTCGAAAGGAGAGGAG	This study

	G	
752	CCGCAAAACAGTTCTTACCTCCTACCGAAGCC	This study
753	AACAGCTATGACCATGATTACGCCAAGCTTGTTAATGGGGGCTAAAGTCGTC	This study
754	AGGTAAGAACTGTTTTGCGGAGAAAGGTTTAACG	This study
755	CGTTGTAAAACGACGGCCAGTGAATTCCTTTGTAAGCGTGCTGAACCG	This study
756	TTGATGCTGCGTCACCTCTATCCCTTTCGTTCC	This study
757	AACAGCTATGACCATGATTACGCCAAGCTTCGTTACCAGACACTTGACGGC	This study
758	TAGAGGTGACGCAGCATCAAAAGGAAGCTCATC	This study
759	CGTTGTAAAACGACGGCCAGTGAATTCGCTGTTGCCAATACGTACGGC	This study
