# Supplementary figures and images for: Use of a microfluidic platform to uncover basic features of energy and environmental stress responses in individual cells of Bacillus subtilis
Source: PLoS Genet. 2017 Jul 20;13(7):e1006901. doi: 10.1371/journal.pgen.1006901 (PMC5542698; doi:10.1371/journal.pgen.1006901)

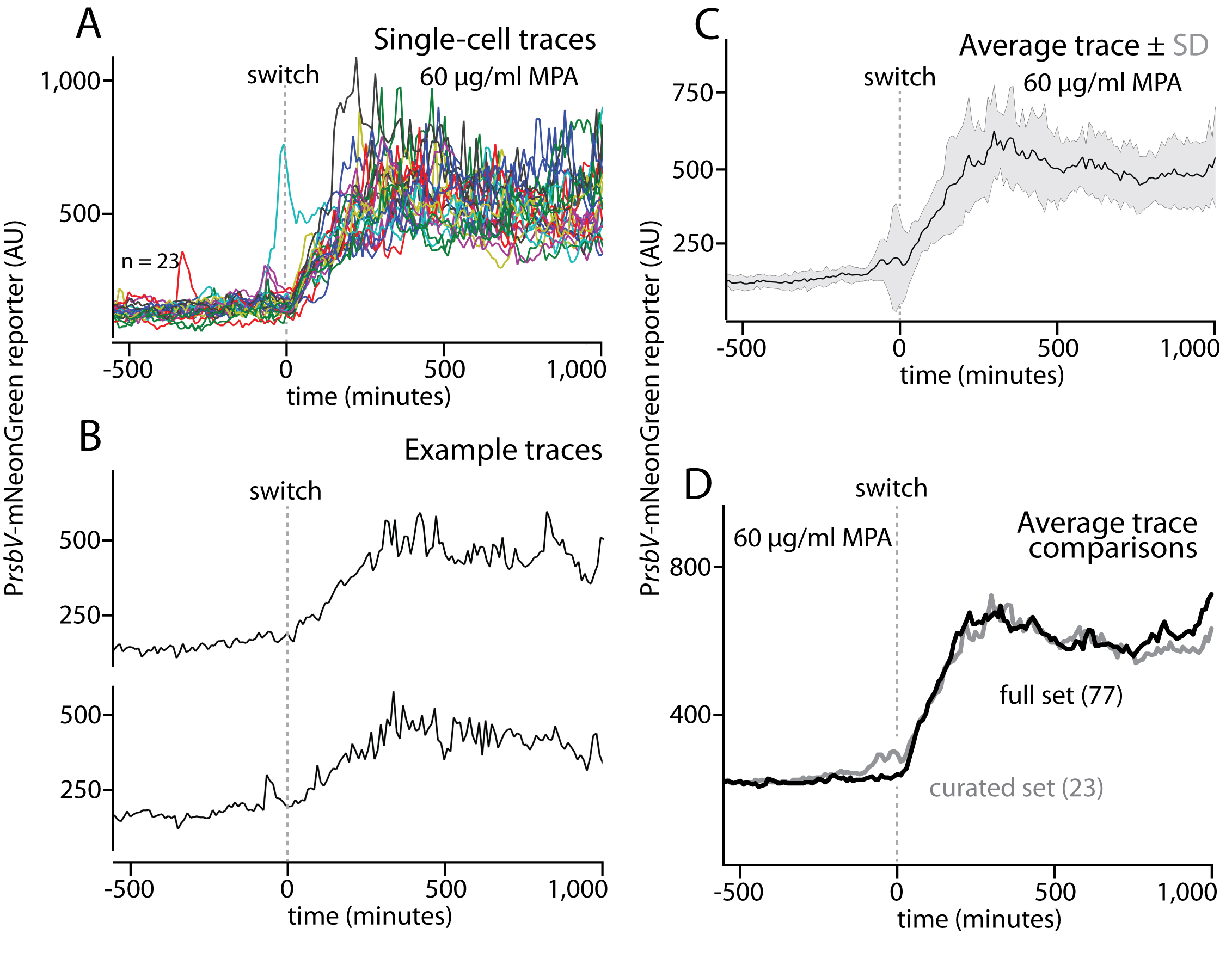

Supplement: S1 Fig — A. Single-cell intensity traces of a stress-responsive PrsbV-mNeonGreen reporter before and after (dashed line) the addition of MPA. B. Two example traces from the ensemble of traces shown in Panel A are shown. C. The average trace from the curated set of cell-lineages shown in Panel A is shown along with the standard deviation (gray envelope surrounding the mean trace). D. Comparison of the average response profile of the full set of lineages from the experiment (black) and the curated set from the same experiment from which lineages displaying cell death, tracking errors, or other artifacts were removed. (TIF) [file pgen.1006901.s002.tif]

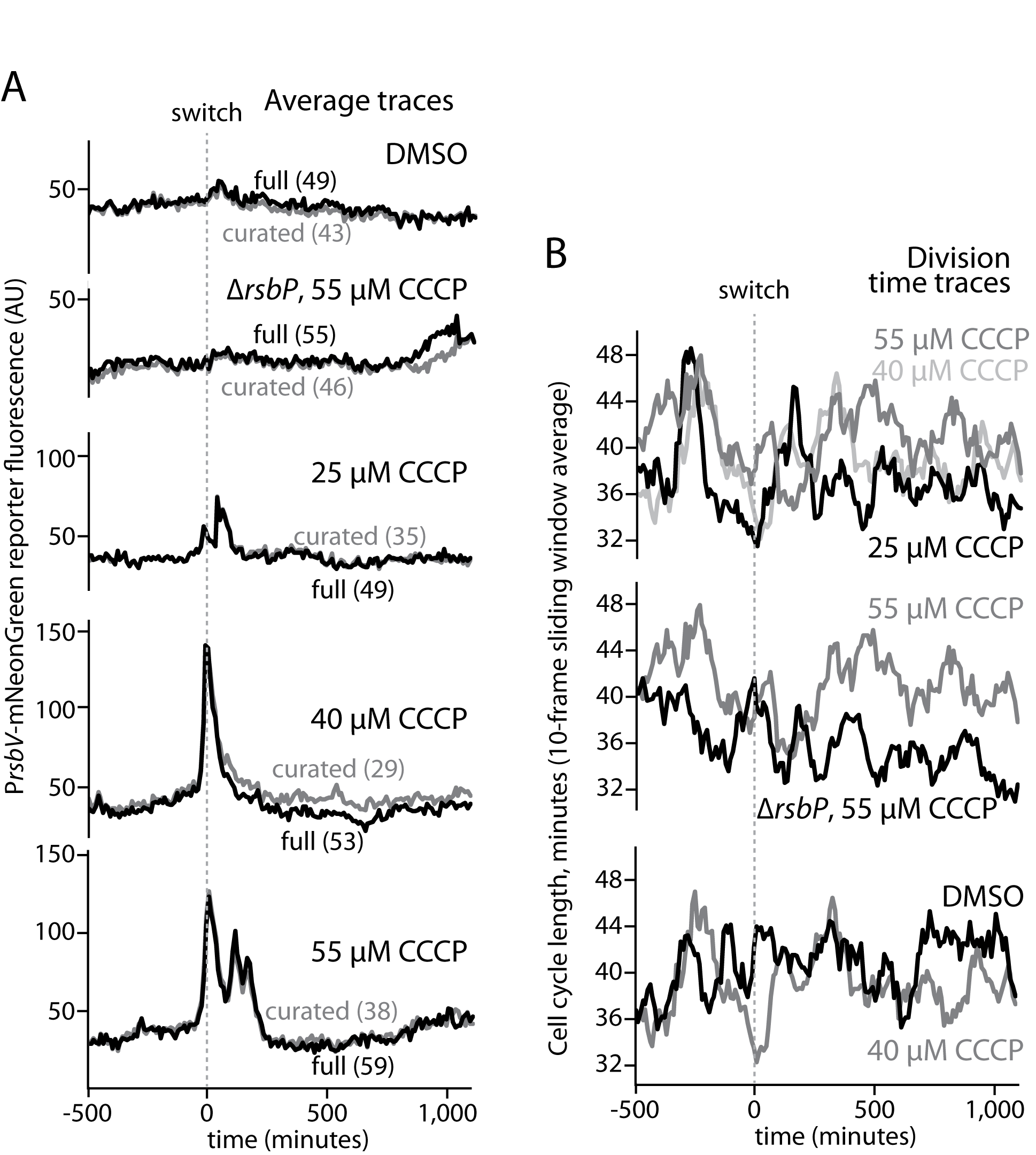

Supplement: S2 Fig — A. Comparisons of the average response profiles from the full set of lineages from a particular experiment (black) and the curated set from the same experiment (gray) from which lineages displaying cell death, tracking errors, or other artifacts were removed. The stress conditions, beginning at the dashed line, are indicated in each plot. B. Comparisons of averaged division times in cell populations experiencing the indicated stress conditions (beginning at the dashed line). (TIF) [file pgen.1006901.s003.tif]

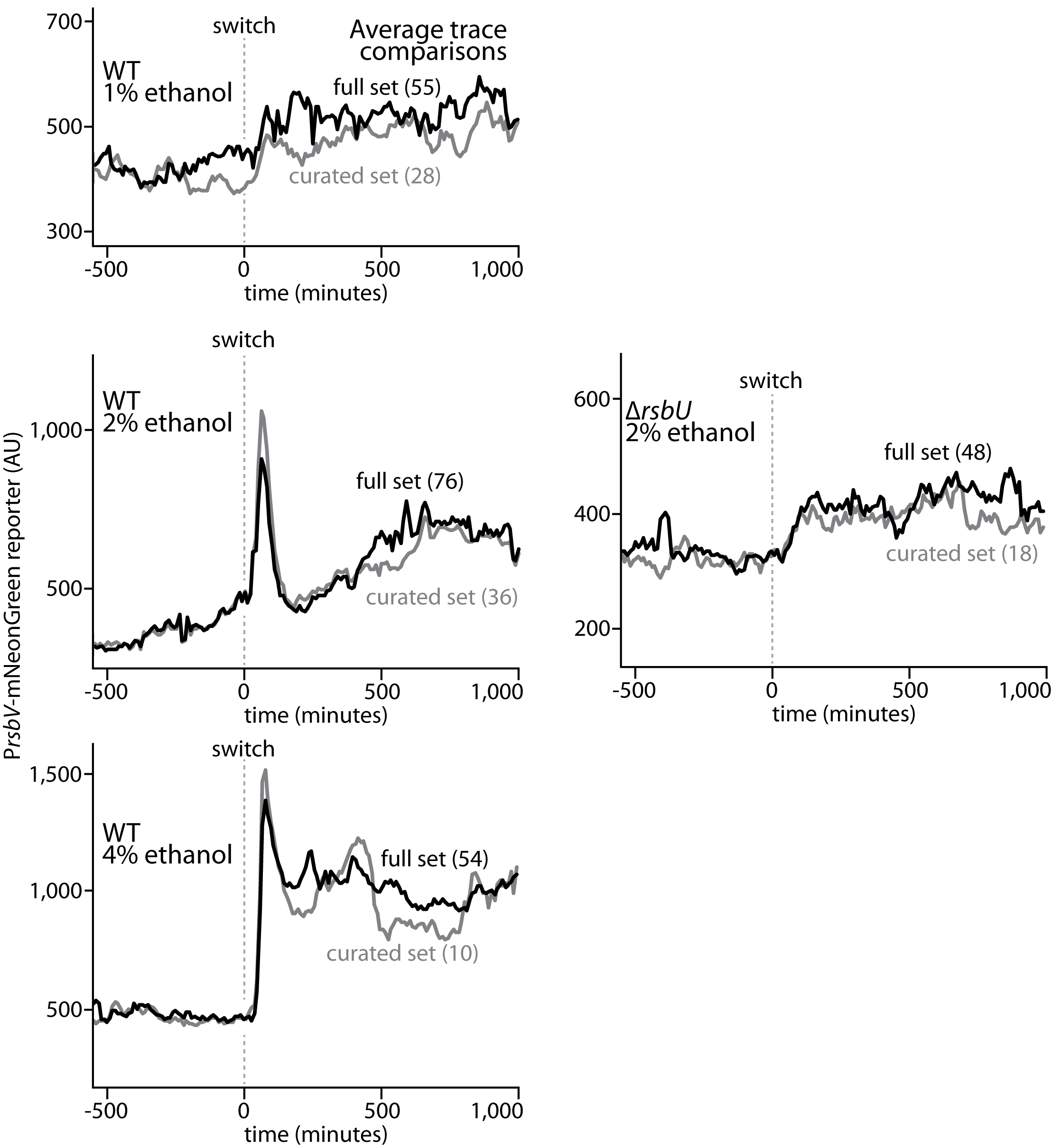

Supplement: S3 Fig — Comparisons of the average response profiles from the full set of lineages from a particular experiment (black) and the curated set from the same experiment (gray) from which lineages displaying cell death, tracking errors, or other artifacts were removed. The stress conditions, beginning at the dashed line, are indicated in each plot. (TIF) [file pgen.1006901.s004.tif]

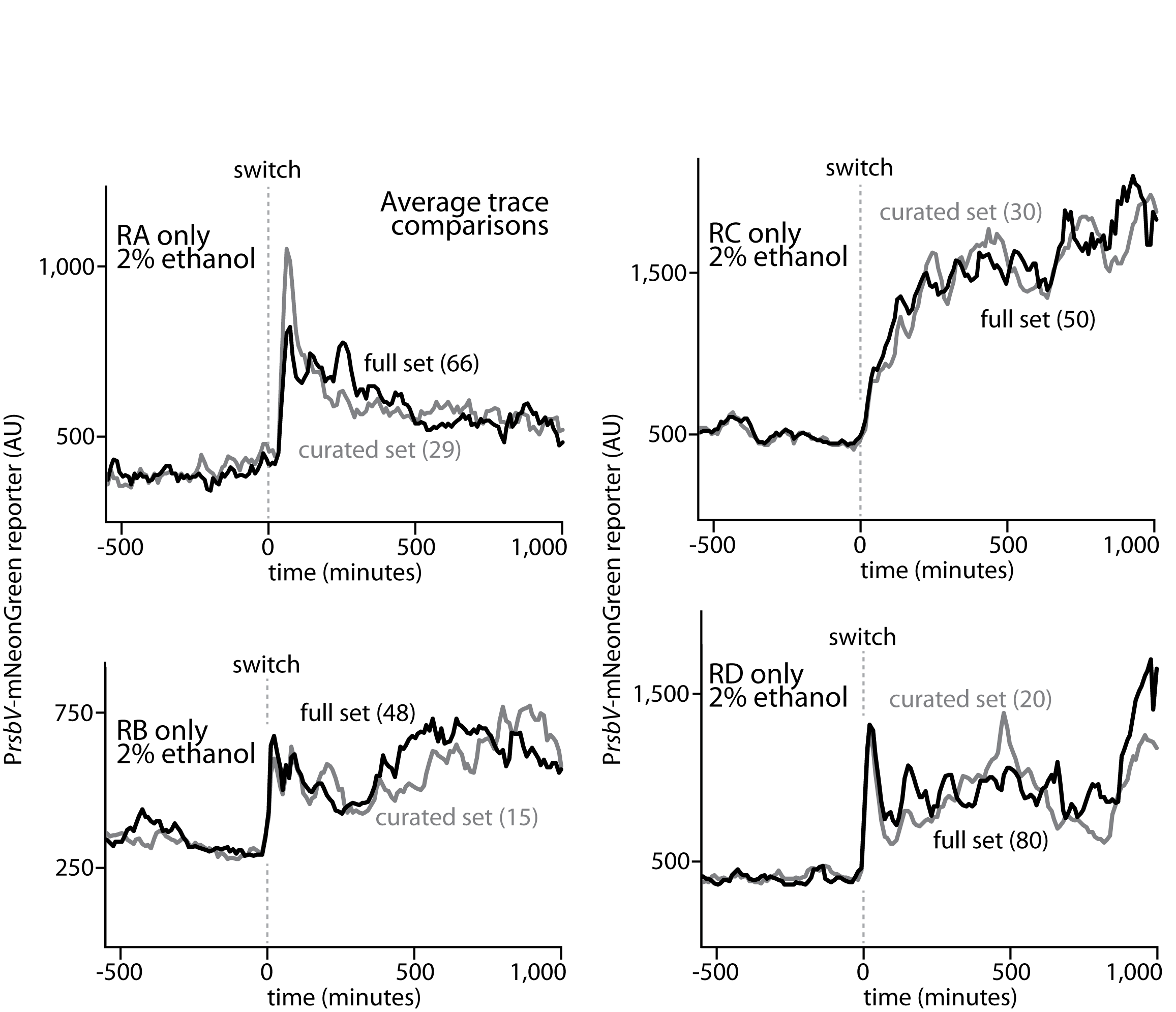

Supplement: S4 Fig — Comparisons of the average response profiles from the full set of lineages from a particular experiment (black) and the curated set from the same experiment (gray) from which lineages displaying cell death, tracking errors, or other artifacts were removed. The strain and stress conditions, beginning at the dashed line, are indicated in each plot. (TIF) [file pgen.1006901.s005.tif]

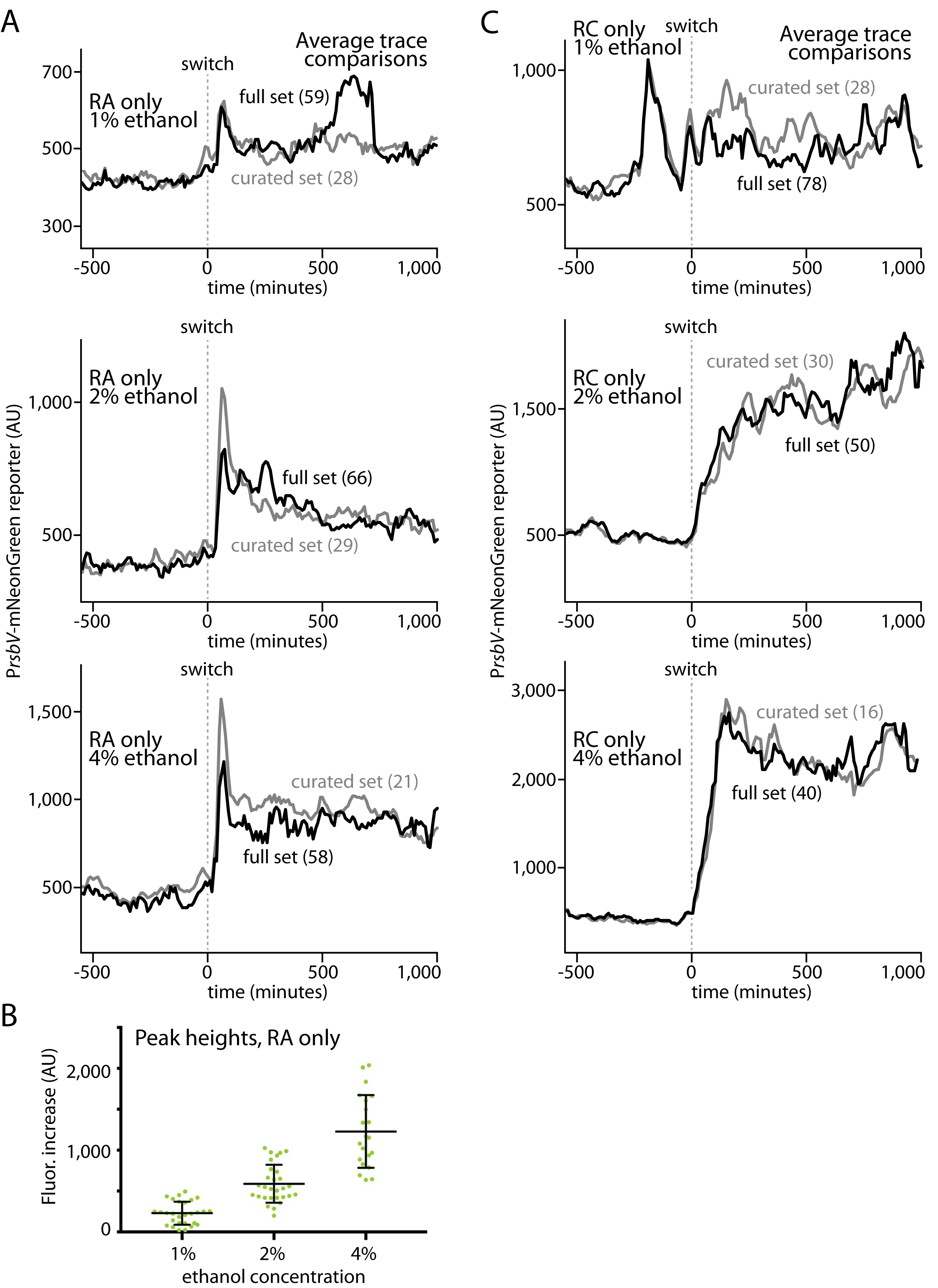

Supplement: S5 Fig — A. Comparisons of the average response profiles of RsbRA-only cells from the full set of lineages from a particular experiment (black) and the curated set from the same experiment (gray) from which lineages displaying cell death, tracking errors, or other artifacts were removed. The strain and stress conditions, beginning at the dashed line, are indicated in each plot. B. Distributions of signal increases in single RsbRA-only cells from before stress exposure to their maximal peak values. All differences were statistically significant (p < 6.8 x 10^−7^). C. Comparisons of the average response profiles of RsbRC-only cells from the full set of lineages from a particular experiment (black) and the curated set from the same experiment (gray) from which lineages displaying cell death, tracking errors, or other artifacts were removed. The strain and stress conditions, beginning at the dashed line, are indicated in each plot. (TIF) [file pgen.1006901.s006.tif]

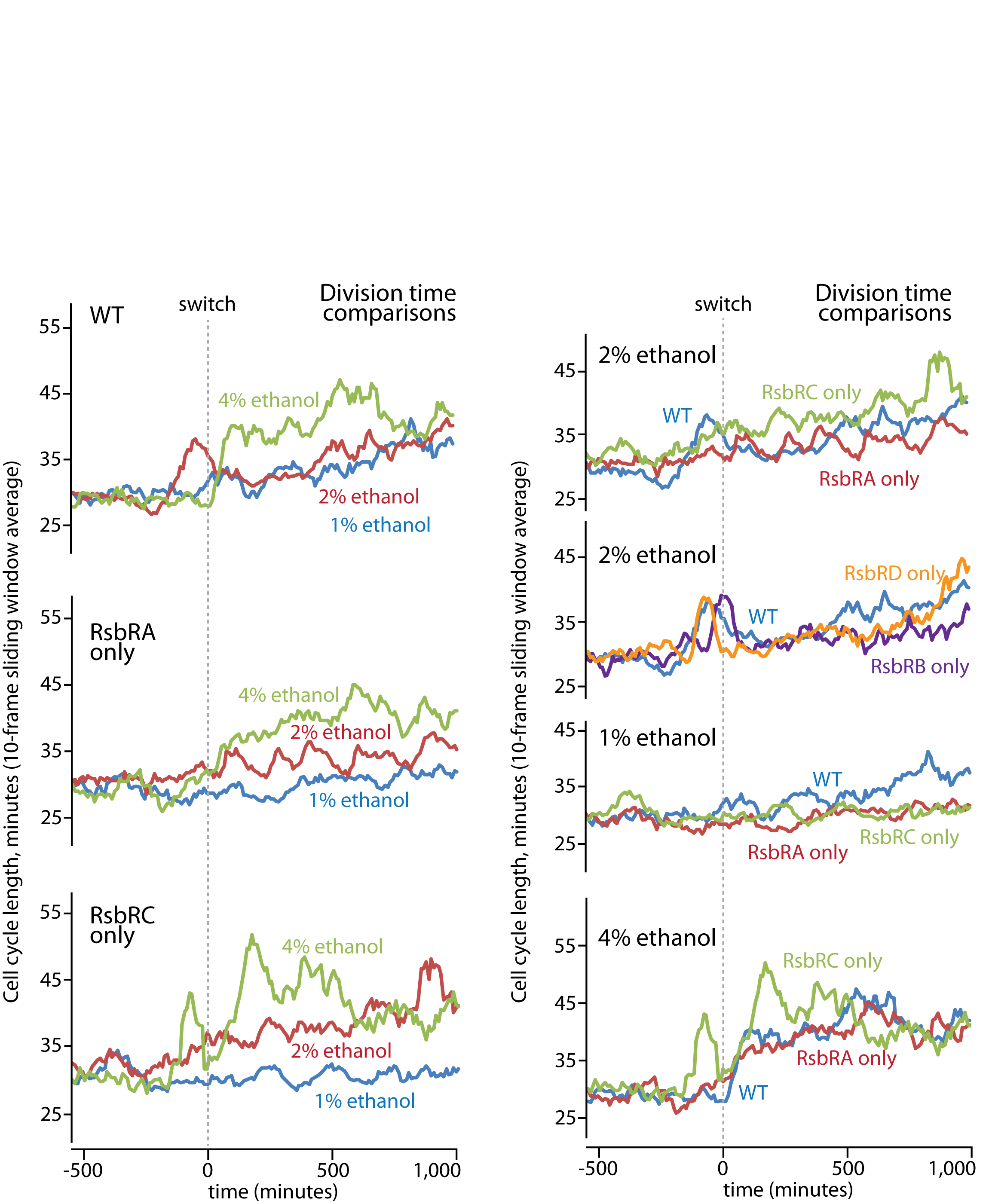

Supplement: S6 Fig — The left panels show overlaid division-time traces at different levels of ethanol stress for the listed strains. The right panels show overlaid division-time traces for different strains as listed in each plot. All traces are averages of a 10-frame sliding window, and the average of the full (uncurated) set of lineages is shown in each case. (TIF) [file pgen.1006901.s007.tif]

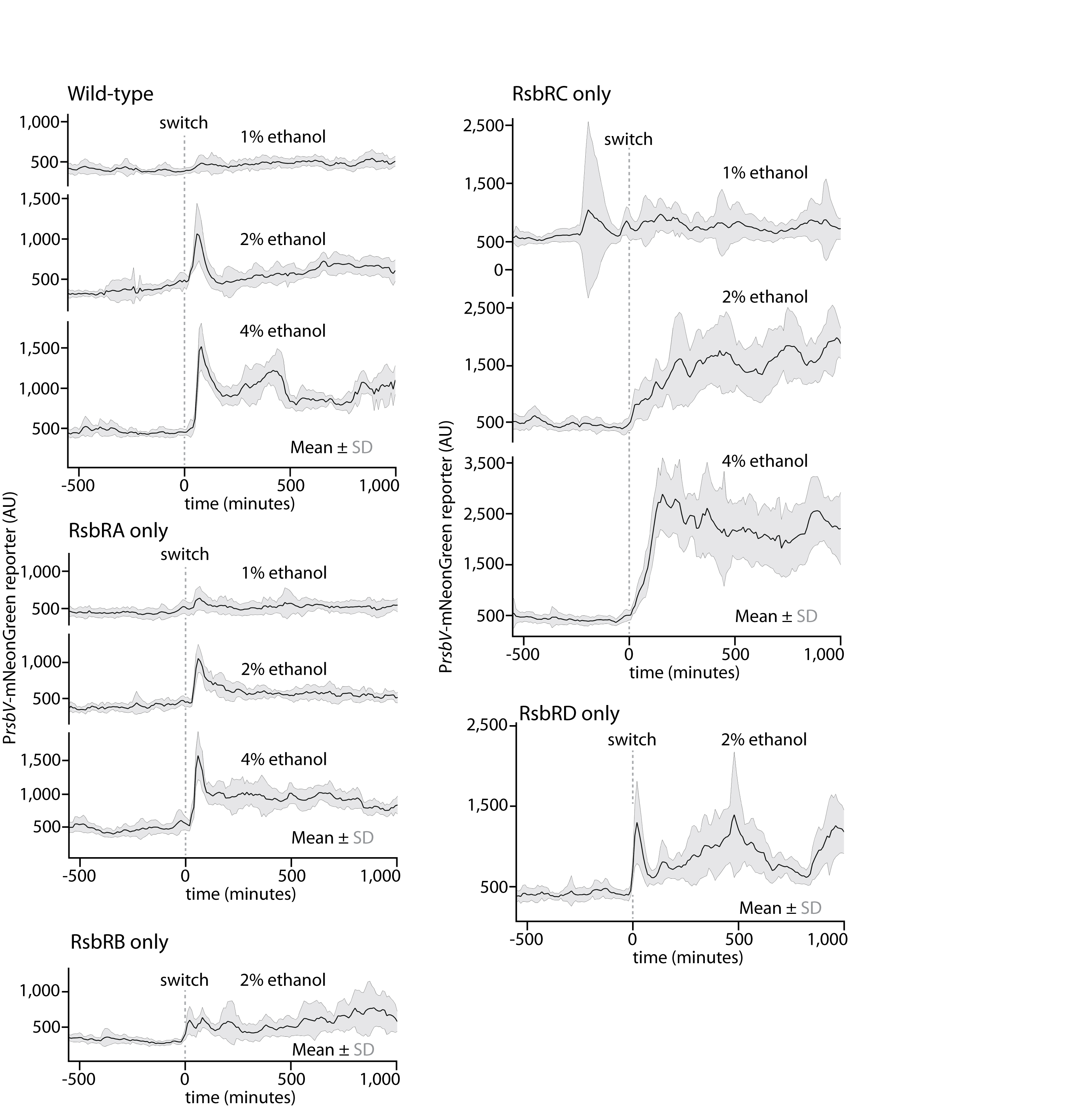

Supplement: S7 Fig — Average traces of the curated cell-lineage sets from the listed strains at the listed ethanol concentrations are shown together with the standard deviation (gray envelope surrounding the mean trace). (TIF) [file pgen.1006901.s008.tif]

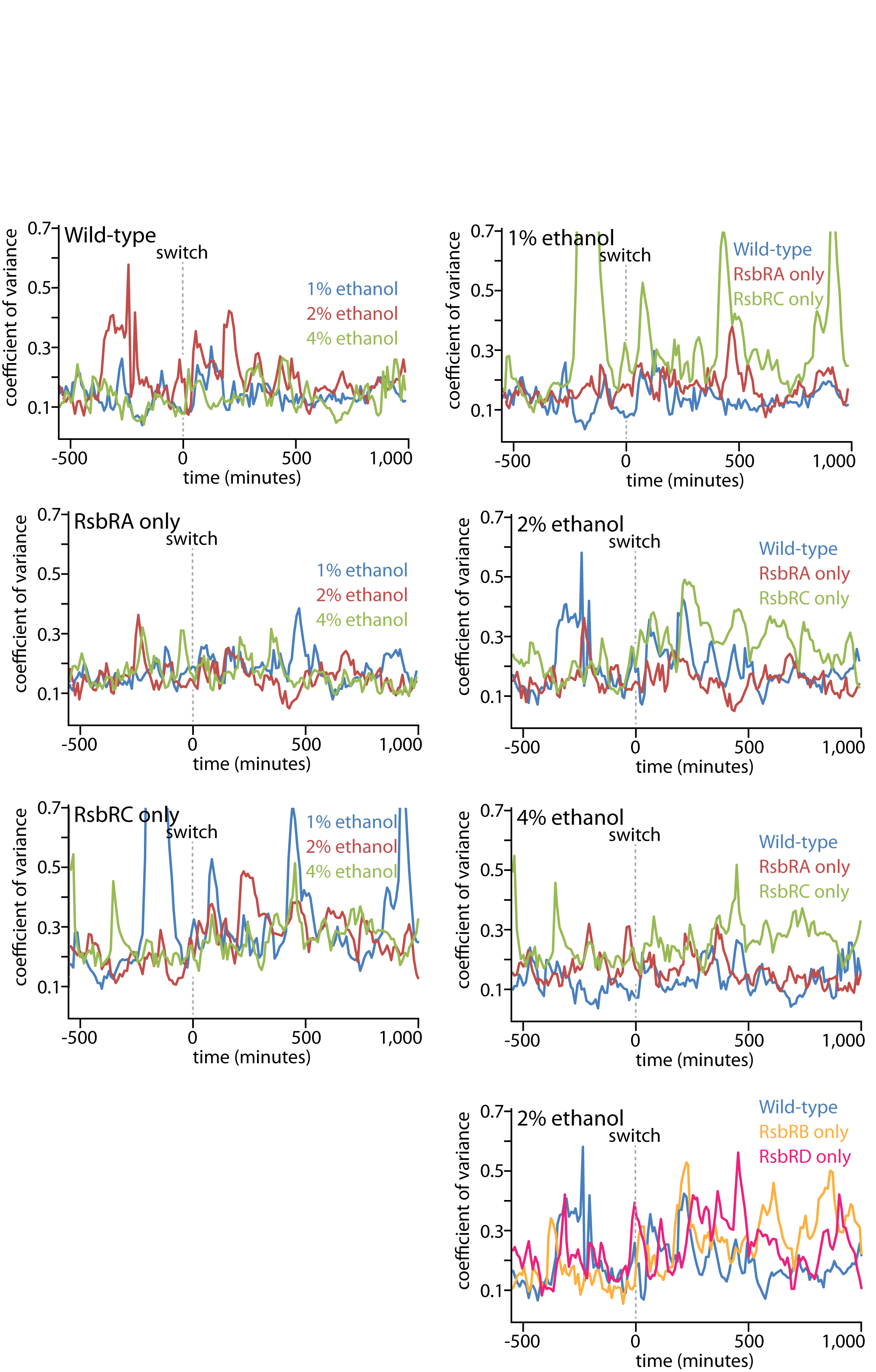

Supplement: S8 Fig — The left panels show overlaid traces at different levels of ethanol stress for the listed strains. The right panels show overlaid traces from different strains at the listed ethanol concentrations. (TIF) [file pgen.1006901.s009.tif]

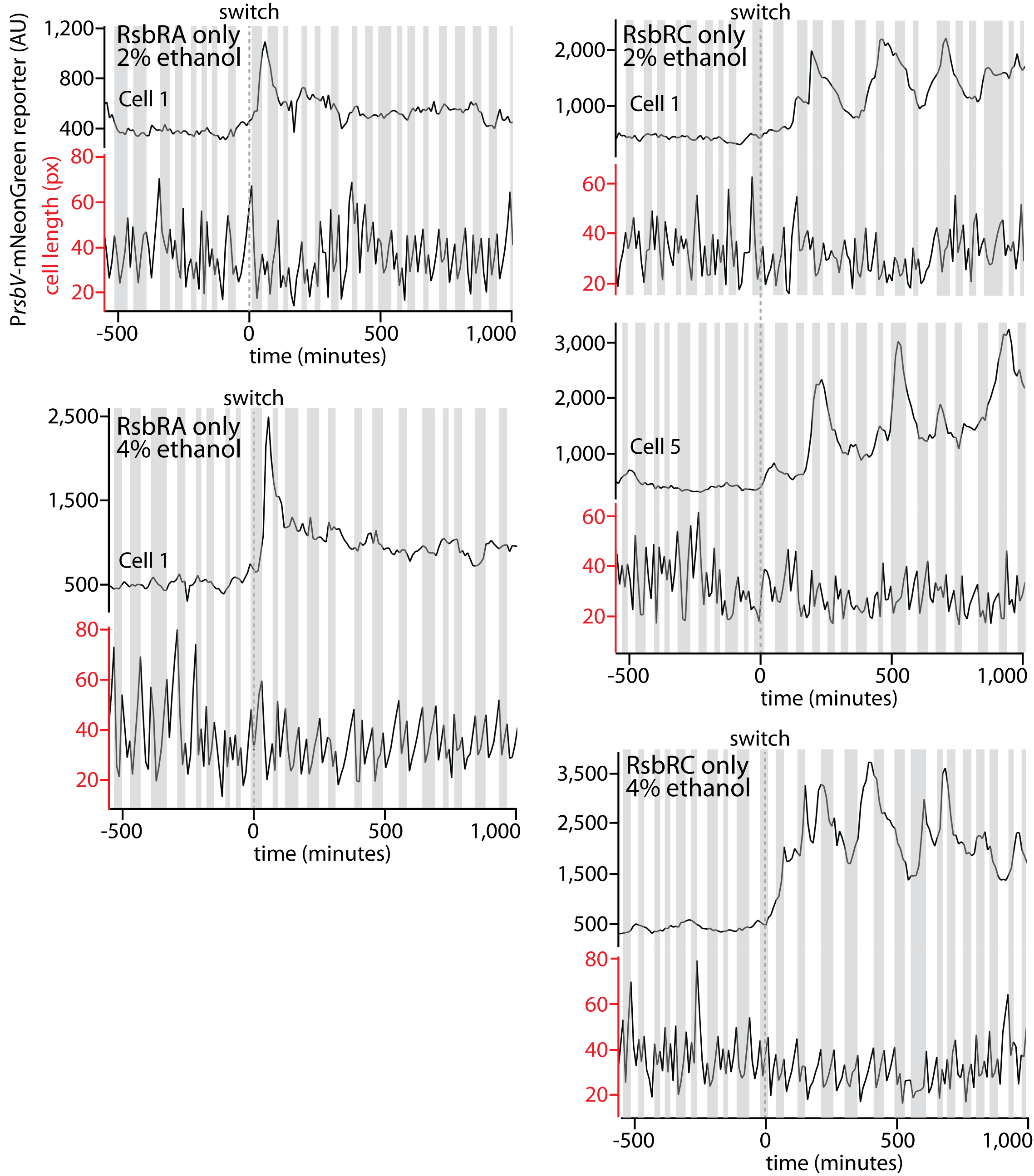

Supplement: S9 Fig — The strain and stress condition for each plot are listed; the example cell numbers correspond to those shown in Fig 5. The top of each plot shows the PrsbV-mNeonGreen reporter intensity, while the bottom of each plot shows cell length, as automatically calculated from constitutive-fluorophore (red, mNeptune) images. For ease of visualization, alternating gray and white bars are placed between consecutive cell-division events, inferred from steep downward slopes in the cell-length traces. (TIF) [file pgen.1006901.s010.tif]

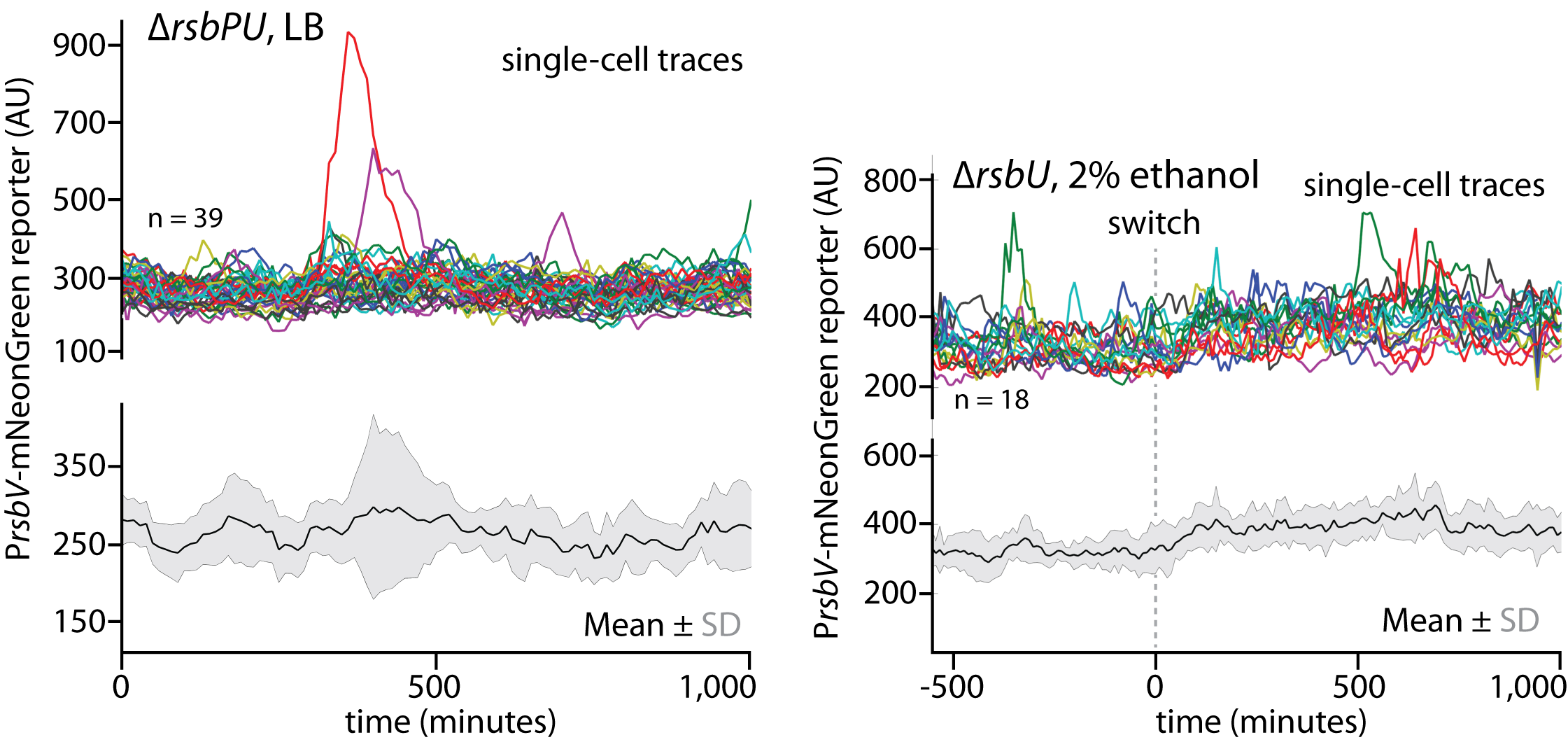

Supplement: S10 Fig — Left panels, response traces of a ΔrsbPU strain (MTC1930) expected to be unresponsive to stress. This experiment was conducted to observe the frequency and magnitude of σB activation events in the absence of both upstream stress-signaling pathways (environmental and energy stress) in unstressed conditions. Right panels, the response to 2% ethanol (dashed line) in ΔrsbU (MTC1920) cells otherwise wild-type for environmental stress (i.e., containing all four RsbR paralogs). The top graphs show overlaid single-cell traces, while the bottom graphs show mean traces with a standard-deviation envelope (gray). (TIF) [file pgen.1006901.s011.tif]

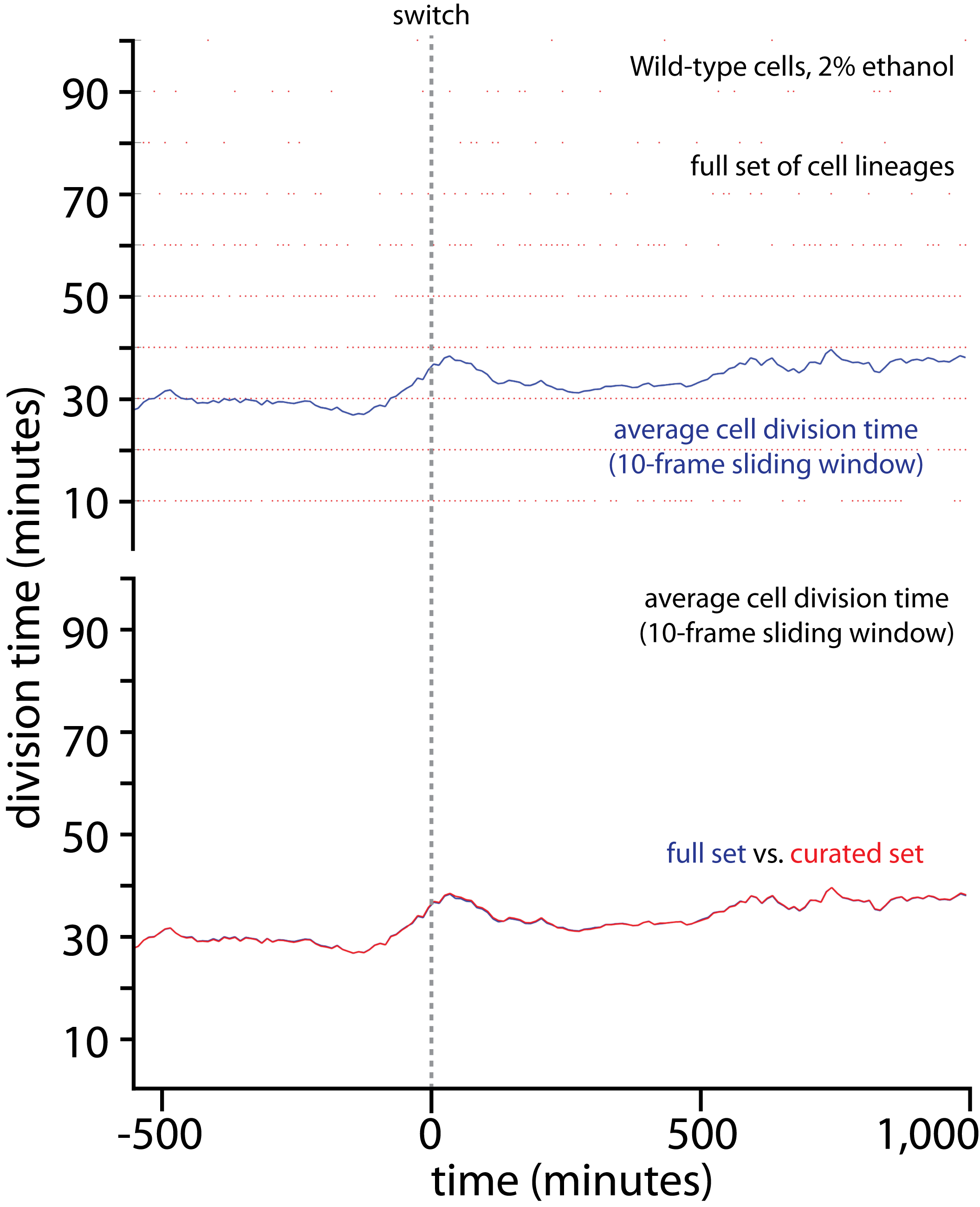

Supplement: S11 Fig — Top panel, a plot of individual-cell division times (dots) along the course of a representative experiment. The time on the Y-axis indicates the time since the last division; the resolution of the method is 10 minutes due to the 10-minute imaging interval used in the experiment. Cell-division events were automatically computed from a constitutive marker (Phyperspank-mNeptune) visible in the red fluorescence channel. The blue trace shows the population division time averaged over a 10-frame sliding window. Bottom panel, a comparison of the average division time plots as computed from the full set of lineages (blue) or the curated set (red) from which lineages displaying cell death, tracking errors, or other artifacts were removed. The traces were indistiguishable in all cases, so we show only the full-set traces in subsequent figures. (TIF) [file pgen.1006901.s012.tif]
